# Evolution of Gene Expression Across Functional Regions of the Mouse Placenta

**DOI:** 10.1093/gbe/evag120

**Published:** 2026-05-23

**Authors:** Fernando Rodriguez-Caro, Emily C Moore, Ashlin Slanger, Jeffrey M Good

**Affiliations:** Division of Biological and Biomedical Sciences, The University of Montana, Missoula, MT 59812, USA; Division of Biological and Biomedical Sciences, The University of Montana, Missoula, MT 59812, USA; Division of Biological and Biomedical Sciences, The University of Montana, Missoula, MT 59812, USA; Division of Biological and Biomedical Sciences, The University of Montana, Missoula, MT 59812, USA

**Keywords:** genomic imprinting, labyrinth zone, junctional zone, maternal decidua, maternal–fetal interface

## Abstract

The eutherian placenta exhibits rapid morphological evolution and is a hotspot for the emergence of reproductive incompatibilities between closely related species. These evolutionary patterns are thought to be a consequence of rapid divergence in gene expression driven by maternal–fetal conflict over resource allocation. However, it remains unclear how the diversity of placental functions shapes gene specialization and expression divergence. We generated genome-wide gene expression and DNA methylation data from fetal and maternal placental tissues of three closely related mouse lineages (*Mus musculus musculus*, *M.’m.’domesticus*, *M. spretus*) and integrated single-cell expression data to investigate how tissue specialization influences gene expression evolution in the rodent placenta. Comparisons among placental regions within *M.’m.’musculus* revealed significant differences in patterns of functional enrichment, imprinting, and X-linked expression across placental layers. The labyrinth zone, the primary site of nutrient exchange, showed strong enrichment for parent-of-origin expression of both autosomal and X-linked genes. Cross-species comparisons of gene expression within each placental layer revealed increased expression level divergence at the maternal–fetal interface. We also identified a subset of genes with maternally biased expression that are spatially associated with the maternal–fetal interface. Parent-of-origin DNA methylation was dominated by epigenetic modification of the maternal genome and interspecific comparisons of parent-of-origin expression revealed overall conservation punctuated by changes in imprinting status of two genes. These findings unveil important links between core elements of placental biology and the evolution of placental gene expression, demonstrating how tissue specialization has influenced parent-of-origin effects and interspecific expression divergence.

Significance In mammals, some genes display silencing of the maternally or the paternally inherited allele, a phenomenon known as genomic imprinting. Imprinting plays a central role in development and has been hypothesized to accelerate molecular evolution in the placenta and contribute to speciation. However, the evolutionary dynamics of placental gene expression and genomic imprinting have remained unclear due to difficulties in identifying imprinted expression. By comparing placental gene expression in three closely related mouse species or subspecies, this study uncovers widespread conservation of imprinting alongside rapid divergence in endocrine-related placental tissues. These results highlight the placental endocrine layer as a major source of interspecific molecular divergence in mammals and provide a framework for understanding how imprinting contributes to placental evolution.

## Introduction

The evolution of a complex mammalian placenta was a key reproductive innovation that enabled prolonged gestation times (Lillegraven et al. 1987, Thompson 1987) and removed critical developmental constraints (Thompson 1987) that propelled extensive morphological innovation across eutherian mammals (Sears 2004; Kelly and Sears 2011). Despite its critical role during development, the complex placenta is among the most rapidly evolving tissues in mammals (Wildman et al. 2006; Chuong et al. 2010), and it is considered a hotspot for the evolution of reproductive incompatibilities between closely related species (Vrana et al. 2000; Zechner et al. 2002; Crespi and Nosil 2013; Brekke et al. 2016). Rapid divergence in the placenta is believed to be fueled, at least in part, by maternal–fetal conflict over resource allocation (Moore and Haig 1991; Babak et al. 2015). However, relatively few placental genes show clear evidence for conflict-driven molecular evolution (Haig 2004; Babak et al. 2015), and the placenta performs several functions beyond nutrient transfer. Thus, it remains unclear how distinct placental functions promote or constrain interspecific molecular divergence.

**Fig. 1.**
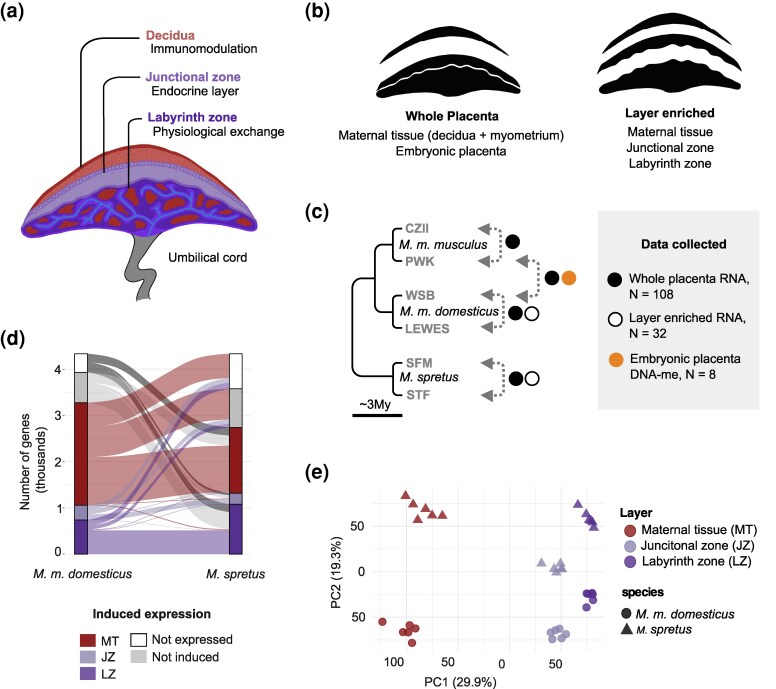
Experimental design and placental gene expression. a) Schematic representation of functional compartmentalization of the rodent placenta. The three major layers include the maternal decidua and the embryonic junctional and labyrinth zones. b) Two types of placenta dissections were performed, including whole placenta dissections with separation of maternal tissue (decidua + myometrium) from embryonic tissues, and layer-enriched dissections with further separation of the two compartments of embryonic portion of the placenta. c) Phylogenetic relations among the three mouse lineages included in this study and data collected from each type of reciprocal cross (see Fig. S1 for sampling details, Supplementary material online). d) Alluvial plot showing overlap in sets of layer-induced genes in *M.’m.’domesticus* and *M. spretus*. e) Principal components analysis of layer-enriched transcriptomes from *M.’m.’domesticus* and *M. spretus* shows clustering of samples by tissue type and species.

Complex mammalian placentas are characterized by remarkable interspecific variation in levels of cellular specialization and more extensive maternal–fetal tissue integration (Blackburn 2015; Roberts et al. 2016) relative to placentation in marsupial mammals and other vertebrate lineages (Stewart and Thompson 2000; Wildman et al. 2006; Blackburn 2015; Furness et al. 2021). Nevertheless, despite considerable morphological variation, the functional architecture of the placenta appears broadly conserved across eutherian mammals (Cross et al. 2003). In most species, the embryonic-derived portion of the placenta is compartmentalized into two major functional layers known as the labyrinth and junctional zones in rodents (Fig. 1a). The labyrinth zone contains a network of fetal capillaries that mediate physiological exchange between maternal and fetal blood (Simmons et al. 2008). The junctional zone is largely made of endocrine tissue that mediates molecular crosstalk between maternal and fetal cells (Simmons et al. 2008; Hu and Cross 2010). In species with more invasive placentation (e.g. rodents and primates), specialized cells derived from the internal lining of the maternal uterine wall form a third placental layer known as the decidua (Abrahamsohn and Zorn 1993). This maternally derived layer plays a critical role in the modulation of the maternal immune response, creating a local uterine environment that prevents rejection of embryonic cells during pregnancy (Wagner et al. 2014; Llorca et al. 2025). Comparative studies have found differences in rates of protein sequence (Chuong et al. 2010) and DNA methylation divergence (Decato et al.’2017) between placental layers in rodents, but layer-specific divergence of gene expression remains uncharacterized.

A unique pattern of gene expression, known as genomic imprinting, is thought to play a major role in eutherian placental function and evolution. Genomic imprinting is an epigenomic phenomenon whereby certain genes show parent-of-origin dependent expression, often determined by epigenetic modifications of gametic DNA (Kelsey and Feil 2013). Throughout development, these epigenetic marks serve as imprinted control regions, influencing silencing of neighboring loci, thus creating clusters of imprinted gene expression in the genome (Barlow and Bartolomei 2014). While only a small number of autosomal genes display imprinted expression in mammals, imprinted genes have large effects on embryonic growth and are highly enriched in placental tissue (Babak et al. 2015; Andergassen et al. 2017; Thamban et al. 2020). Enrichment of imprinted expression in placental tissue is particularly strong in rodents, where the paternal X chromosome is silenced, resulting in predominantly maternal expression of genes on the X chromosome (Takagi and Sasaki 1975). In parallel, mammal hybrids often show parent-of-origin growth effects, which have been linked in rodents to disruption of imprinted gene expression (Vrana et al. 2000; Zechner et al. 2002; Brekke et al. 2016) and DNA-methylation (Arévalo et al. 2021), as well as misregulation of large imprinted gene networks (Brekke et al. 2021). These findings suggest rapid accumulation of regulatory incompatibilities at imprinted loci that play critical roles in placental development. However, the relative contribution of imprinted genes to overall expression divergence has not been quantified in the rodent placenta.

House mice (*Mus musculus* and related species) provide a powerful system to study the evolution of placental gene expression in mammals. *Mus musculus* includes three subspecies—*M.’m.’musculus*, *M.’m.’domesticus*, *M.’m.’castaneus*—which arose within the last 500,000 years (Phifer-Rixey et al. 2020; Agwamba and Nachman 2023; Agwamba et al. 2025). This group provides extensive genomic variation (Phifer-Rixey et al. 2014) and a high-quality reference genome (Waterston et al. 2002; Church et al. 2009). Moreover, several wild-derived inbred strains have been extensively genotyped, providing an opportunity to leverage the genetic diversity of this genus (Keane et al. 2011; Chang et al. 2017; Srivastava et al. 2017). Previous studies on placental gene expression provide powerful information on the mouse placental imprintome (Wang et al. 2011; Okae et al. 2012; Finn et al. 2014; Andergassen et al. 2017) and single-cell expression patterns (Marsh and Blelloch 2020; Jiang et al. 2023; Wu et al. 2024; Stadtmauer et al. 2025). Together, these resources provide an opportunity to advance our understanding of placental molecular evolution.

In this study, we evaluate the central hypothesis that the evolution of placental gene expression is shaped by maternal–fetal conflict over maternal resource allocation, with imprinted genes acting as key mediators of this conflict. We test two predictions related to this overarching hypothesis. First, if placental imprinting has evolved primarily in response to maternal–fetal conflict over the regulation of placental nutrient-transfer capacity, then signatures of autosomal and X-linked imprinting should be enriched within the labyrinth zone, the layer dedicated to nutrient transfer. Second, if such conflict also drives rapid evolution of the placenta, then signatures of molecular evolutionary divergence should also be more pronounced in the labyrinth zone and disproportionally involve imprinted genes. To evaluate these predictions, we generated gene expression and DNA methylation data from functionally distinct placental layers using crosses between six wild-derived mouse strains spanning two subspecies of *M. musculus* (*M.’m.’musculus* and *M.’m.’domesticus*) and the closely related Algerian mouse, *M. spretus*. We analyzed these data using a custom analytical model developed to minimize the contribution of maternal contamination in estimates of gene expression of placental tissues. We then integrated newly available single-cell expression data (Wu et al. 2024) to ask how specialization across placental tissue layers and cell types shapes genome-wide patterns of gene expression, imprinted expression, and interspecific divergence.

## Results

We used a series of experimental crosses between strains and subspecies of house mice to study the functional specialization and evolution of placental expression (Fig. 1b and c; Fig. S1, Supplementary material online). Our experimental design included three key elements. First, we used layer-enriched placental tissue sampling to understand patterns of gene expression across functionally distinct regions of the placenta and maternal versus embryonic contributions to placental transcriptomes. Second, we used reciprocal crosses within and between different subspecies of house mice (*M. musculus*), pairing whole embryonic placenta with maternal placental tissue dissections to develop a model of imprinted gene expression that accounts for maternal tissue contamination. We complemented this expression dataset with whole-genome DNA methylation data to assess parent-of-origin patterns of placental methylation and to further evaluate our placental imprinting model. Finally, we applied our analytical model to additional whole and layer-enriched placental data from a closely related species, *M. spretus*, to begin to understand evolutionary conservation and divergence of placental imprinting between mouse species.

### Patterns of Gene Expression Across Placental Layers Reflect Functional Specialization

To identify genes associated with placental tissue specialization, we first quantified expression from 36 layer-enriched dissections from *M.’m.’domesticus* and *M. spretus* (six junctional zone, six labyrinth zone, six maternal tissue per species). After read filtering, RNA-seq libraries showed an average depth of 14.8 million reads per sample. Principal component analysis showed clustering of samples by tissue type along PC1 (29.9% variance explained), then by species origin along PC2 (19.3% variance explained; Fig. 1e). We detected 15,594 genes expressed (zFPKM > −3) in at least one layer of the *M.’m.’domesticus* placenta, of which 3,272 (20.9%) showed a 2-fold increase in one of the layers relative to the others (i.e. layer-induced expression following Kopania et al. (2022)). In *M. spretus*, 15,422 genes were expressed, including 2,738 (17.8%) that were layer-induced (Tables S1 and S2, Supplementary material online). When considering all genes expressed in placental tissues, species showed high overlap of placentally expressed genes, with 15,247 genes (94.6%) expressed in both species, 347 (2.1%) only in *M.’m.’domesticus*, and 519 (3.2%) only in *M. spretus*. In both species, the maternal tissue showed the largest number of layer-induced genes, followed by the labyrinth zone and the junctional zone (Fig. 1d).

We validated our approach for identifying layer enrichment in expression by comparing our layer-induced gene sets to two published single-cell RNA-seq (scRNA-seq) expression datasets (Jain and Tuteja 2021; Wu et al. 2024). We found significant enrichment for layer-specific gene markers among layer-induced gene sets (Fig. S2 a, c, e, Supplementary material online) and high expression bias scores in the expected cell types (Fig. S2 b, d, f). Of note, genes induced in maternal tissue also showed enrichment for markers associated with embryonic cells, likely due to the presence of invasive trophoblast cells in maternal tissue.

Sets of layer-induced genes showed substantial overlap between species, but this overlap varied by layer. After excluding genes with very low expression (zFPKM < −3) in either species, 65.8% of genes induced in *M.’m.’domesticus* also showed induced expression in the same layer in *M. spretus*. The highest similarity was observed in the labyrinth zone (84.9%), followed by the maternal tissue (59.4%) and the junctional zone (48.6%). Because these comparisons depend on a categorical variable (i.e. induced or not induced), we re-evaluated these overlaps using a continuous measure of induced expression and found that 6% to 12% of the discordance between species could be attributed to threshold effects (Fig. S3, Supplementary material online). More generally, scores of layer-induced expression between species were highly correlated transcriptome-wide (*R* = 0.79, maternal tissue; *R* = 0.80, labyrinth zone; *R* = 0.70, junctional zone; Pearson's correlation, all *P* ≪ 0.001).

Functional enrichment analysis among sets of layer-induced genes supported overrepresentation of predicted placental functions of all layers in both *M.’m.’domesticus* and *M. spretus*. For example, 68% of REACTOME-enriched terms among decidua-induced genes were immune-related with “immunoregulatory interactions between lymphoid and nonlymphoid cells” (*P* = 3.49e−17 in *M. m domesticus*; *P* = 0.002 in *M. spretus*) and “complement and coagulation cascades” (*P* = 2.3e−7 in *M.’m.’domesticus*; *P* = 1.58e−12 in *M. spretus*) among the top significantly enriched terms. In the junctional zone, seven of the eight terms (87.5%) were related to signal transduction, including “class A/1 (Rhodopsin-like) receptors” (*P* = 3.15e−07 in *M.’m.’domesticus*) and “neuroactive ligand–receptor interactions” (*P* = 6.05e−07 in *M.’m.’domesticus*; *P* = 0.04 in *M. spretus*). Lastly, 11 of the 13 terms enriched among labyrinth-zone-induced genes (84.6%) were associated with nutrient transport, including “vitamin digestion and absorption” (*P* = 8.8e−04 in *M.’m.’domesticus*) and “SLC-mediated transmembrane transport (*P* = 6.01e−4 in *M.’m.’domesticus*; *P* = 3.36e−03 in *M. spretus*).” A complete list of functional terms enriched on each gene set is provided in Table S3 (Supplementary material online). Collectively, these results identify genes with expression that is tightly associated with specific placental tissues, establishing a framework for downstream analysis of gene expression and imprinted expression evolution.

### The Imprinted Gene Expression Landscape of the Mature House Mouse Placenta

We next focused on the identification of imprinted expression in the mature placenta, including imprinted X chromosome inactivation. For this, we integrated fetal and maternal expression data from reciprocal crosses between *M. musculus* strains (Fig. 1c) to create a model of *M. musculus* placental imprinted expression (i.e. genes showing strong parent-of-origin expression bias) while using a custom modeling framework to account for the effects of maternal contamination and other sources of error (see Methods).

Our initial (uncorrected) screen detected parent-of-origin expression bias for a total of 292 autosomal genes in at least one of the three reciprocal cross’types’(135 genes in the cross between *M.’m.’domesticus* strains; 128 genes in the cross between *M.’m.’musculus* strains; 235 genes in the cross between *M. musculus* subspecies). Differences in the number of genes detected were consistent with differences in power for discerning parental alleles based on diagnostic nucleotide variants’between strains of each cross type (Table S4, Supplementary material online). Our model for maternal contamination estimated a median sample contamination (percent of maternal tissue present on’embryonic placenta samples) of 6.9% (Fig. S4, Supplementary material online). Therefore, we adjusted parent-of-origin bias scores accounting for background contamination at each’gene using cross-specific estimates of maternal contamination and gene expression levels in contaminating maternal cells (See supplementary methods, Supplementary material online). To further minimize the effect of maternal contamination, we excluded maternally biased genes without validated imprinted status in the mouse literature if (i) they were predominantly expressed in blood (i.e. an additional source of contamination directly accounted for in our model) using data from the bloodExpress database (Miranda-Saavedra et al. 2009), or (ii) didn’t show significant parent-of origin expression under a more conservative cut-off of contamination (Fig. S5, Supplementary material online).

Several metrics supported effective transcriptome-wide removal of maternal contamination effects using this analytical approach (Fig. S6, Supplementary material online). Our final gene set included 56 autosomal genes showing parent-of-origin expression, including 38 genes previously validated and 18 novel candidate genes for imprinted expression (Table S5, Supplementary material online). Maternally versus paternally biased genes were equally represented among the 38 previously validated autosomal genes (19 maternally biased genes, 19 paternally biased genes; Fig. S7a, Table S5, Supplementary material online), consistent with some previous results (Wang’et al. 2011; Okae et al. 2012). In contrast, the 18 novel candidates all showed maternally biased expression (Fig. S7b). On the X chromosome, 409 genes showed maternally biased expression, 15 genes showed’biallelic expression, and the noncoding RNA *Xist* showed paternally biased expression, all consistent with a regulation landscape dominated by imprinted X-chromosome inactivation (Takagi and Sasaki 1975)’with some genes escaping paternal imprinting (Ma’et al 2018).

### Large Contributions of Maternal DNA-methylation at Imprinted Loci

Next, we complemented our gene expression-based model with an analysis of allele-specific DNA methylation in whole fetal placenta tissues of *M. musculus*. Placental tissues showed an average of 49.7% CpG methylation, consistent with previous studies (Decato et al. 2017). We observed higher levels of DNA methylation of the maternal genome (50.94%) compared to the paternal genome (48.9%, Welch *t*-test, *P* ≪ 0.01). Of 3,932 parent-of-origin differentially methylated regions (DMRs), 3,745 (95.2%) showed maternal DNA methylation (Table 1, Table S6, Supplementary material online). This strong excess of maternal DMRs was aligned with previous reports from rodents and humans (Kobayashi et al. 2012; Hanna et al. 2016; Richard Albert et al. 2023). However, to ensure that the estimated maternal bias was not due to maternal contamination, we removed 6.8% of CpG methylation-supporting maternal read counts—corresponding to the estimated average sample contamination and conservatively assuming 100% DNA methylation of contaminating reads. After correction, 83% of CpG sites responsible for the excess of maternal methylation remained biased toward the maternal genome, indicating that maternal tissue contamination was insufficient to explain the large excess of maternal DNA-methylation observed.

**Table 1:** Contribution of maternal and paternal DNA methylation to the parent-of-origin DNA methylation landscape of the *M. musculus* placenta

Origin of DNA methylation	Total DMRs	Average DMR length (bp)	DMRs linked to genes	DMRs linked to regulatory elements	DMRs linked to imprinted genes
Maternal	3,745	11,157	1,470	2,738	26
Paternal	186	1,642	69	53	3

Parent-of-origin DMRs linked to imprinted genes were predominantly defined by maternal DNA methylation. We detected 29 DMRs linked (within 2 kb) to genes showing imprinted expression, of which 27 (93%) were maternally methylated. Notably, maternal DMRs were linked to both maternally and paternally biased genes (7 and 10 genes, respectively; Fig. S7a). These results support the extensive contributions of the maternal genome to parent-of-origin DNA methylation in the mouse placenta and, likely, the regulation of imprinted loci (Wolf and Hager 2006). We also observed a higher association with parent-of-origin DMRs among experimentally validated versus novel candidate genes for imprinted expression. Of the 38 genes with validated imprinted status, 17 (44%) were physically linked to a DMR (i.e. within 2 kb, Fig. S7c, Supplementary material online) and 13 (34%) were linked to a differentially methylated imprinting cluster (Figs. S5, Supplementary material online). In contrast, only two of the 18 unvalidated candidates were physically linked to parent-of-origin DMRs (*Mamdc2* and *Adamts5*; 11%), and another (*Mrgprg*; 5%) was nested in the differentially methylated *Kcnq1* cluster. This pattern may reflect persistence of false positives among the novel candidates, contributions of DNA-methylation independent (noncanonical) imprinting, or a combination of both.

### Maternally Biased Expression Reveals Genes Strongly Associated With the Invasive Trophoblast Interface

Our correction for maternal contamination was effective to remove genome-wide effects of maternal contamination (Fig. S6, Supplementary material Online), but all of our novel candidates for imprinted expression showed maternally biased expression and a low prevalence of parent-of-origin DMRs. In principle, these genes could represent novel imprinted genes or reflect a form of contamination not accounted for by our contamination model. For example, highly specific expression from specialized maternal cells is localized within the junctional zone or at the interface between the junctional zone and the maternal tissue. This form of maternal cell contamination (spatially localized and cell-type specific) would likely evade model-based correction from bulk RNA-seq data. However, such interface-specific genes are likely to play a critical role in placental biology through regulation of trophoblast invasion (Clark et al. 2002, Stadtmauer et al. 2025) regardless of imprinting status. To further explore the source of maternally biased expression in our candidates, we performed an RNA fluorescence in situ hybridization (FISH) experiment to localize spatial placental expression of three candidate genes (*End2*, *Tnfrs11b*, *Erv3*). If maternal bias was a product of unaccounted contamination, we predicted expression in maternal tissue at the maternal–fetal interface. Consistent with our predictions, all three showed expression primarily from maternal tissue near invasive trophoblast cells (Fig. S8, Supplementary material online). We refer to this narrow region of the junctional zone as the interface of invasion, following Wu et al (2024).

**Fig. 2.**
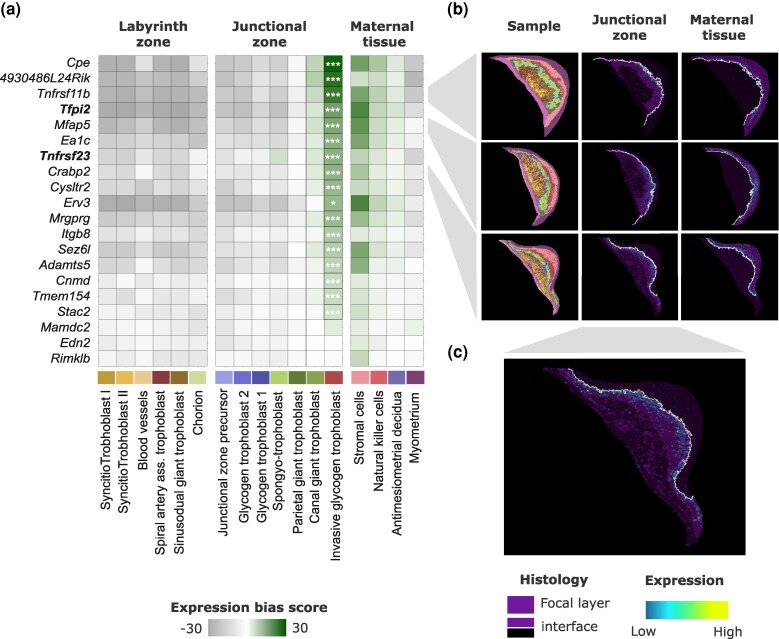
Evaluation of spatial patterns of expression of novel candidates for imprinted expression. a) Expression bias scores of 18 candidates and two known imprinted genes (in bold) at single-cell resolution from Wu et al (2024). Cell types are grouped by layer. Stars mark significance of DE test of expression in invasive trophoblast versus all other cell types (* P < 0.05., *** P < 0.001). b) Spatial patterns of expression of three candidates. The first column shows distribution of cell subtypes across three placenta samples following color scheme in (a). Second and third columns show expression of focal genes in the junctional zone and maternal tissue, respectively. Focal layer is colored in dark purple, nonfocal layer in black and the invasive trophoblast interface (juxtaposition of embryonic and maternal cells) is marked with a dotted white line. c) Expanded image of the expression pattern of *Crabp2*, showing expression in fetal cells.

We next used spatially resolved placental scRNA-seq data (Wu et al. 2024) to inspect spatial expression patterns of all 18 novel candidates and test for association with this interface. In addition to our 18 novel candidates, we included two genes (*Tnsfrsf23* and *Tfpi2*) whose expression had been previously associated with the interface of invasion (Clark et al. 2002; Okae et al. 2012) as positive controls. Single-cell data confirmed an association with the interface of invasion but revealed a more complex expression pattern. Most genes tested (16 of 20) showed significant upregulation in invasive trophoblast (fetal), and 12 of these were expressed by interface-associated maternal cells (Fig. 2a). Visual inspection of expression patterns confirmed that most of these genes were specifically upregulated in fetal and maternal cells that are in direct contact with the interface of invasion (Fig. 2b and c). Functional characterization of this gene set showed several associations with cell signaling, immunosuppression, and growth restriction (Table S7, Supplementary material online). Together, these spatial and functional patterns suggest a role in the regulation of trophoblast invasion, vascularization, and/or implantation signaling. While these results do not directly address the imprinted status of these candidates, they suggest that imprinting is not necessary to explain maternally biased expression while also revealing co-expression between maternal and fetal cells for a small set of genes at the interface of invasion. Given these considerations, we excluded these novel candidates with maternally biased expression from downstream analysis of imprinted expression.

### The Labyrinth Zone is Strongly Enriched for Imprinted Autosomal and X-linked Expression

Having refined our gene sets of layer-induced and imprinted expression in the *Mus musculus* placenta, we asked whether genomic imprinting is associated with tissue specialization in the placenta. Under the hypothesis that imprinting evolves primarily in response to conflict over maternal resource allocation, we predicted that imprinted genes would be overrepresented among genes induced in the labyrinth zone, the layer specialized in nutrient transfer.

**Fig. 3.**
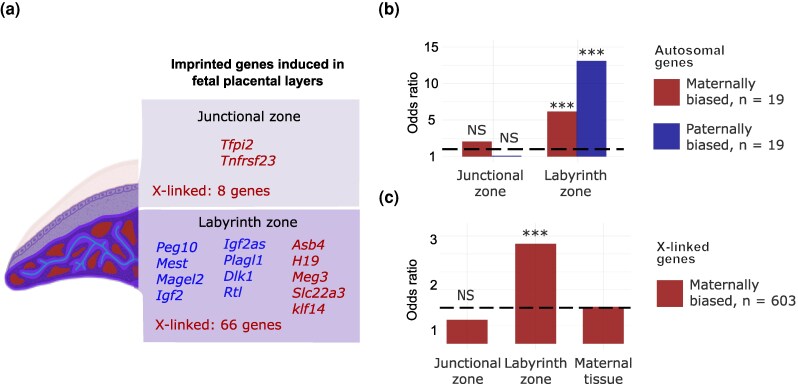
Spatial expression patterns of imprinted genes in the mouse placenta. a) Autosomal imprinted and X-linked genes showing layer-induced expression in embryonic layers of the *M. musculus* placenta. Results from overrepresentation test of b) autosomal imprinted and c) X-linked genes among genes induced in placental layers (hypergeometric, NS: not significant, *P < 0.05, **P < 0.01, ***P < 0.001).

To evaluate this prediction, we tested for significant enrichment of imprinted genes among sets of layer-induced genes. We found 15 autosomal imprinted genes (Fisher's exact test, *P* = 5.19e−08) and 74 maternally biased X-linked genes (Fisher's exact test, *P*’= 9.47e−08) induced in embryonic placenta layers. Consistent with our prediction, this enrichment was largely driven by labyrinth zone tissue where 13 autosomal genes (Fisher's exact, *P* = 8.04e−06) and 66’X-linked genes (Fisher's exact, *P* = 2.4e−11) were identified as layer-induced (Fig. 3). In contrast, genes induced in the junctional zone did not show enrichment for imprinted expression (*N* = 2 genes, Fisher's exact, *P*’= 0.23). These striking differences in the prevalence of imprinted expression among layer-induced genes suggest a close association between the evolution of genomic imprinting and labyrinth zone tissue specialization. Notably, while this includes enrichment for both maternal (Fisher's exact, *P* < 0.01) and paternal autosomal imprinting (Fisher's exact *P* ≪ 0.001), maternally biased expression was far more abundant in the labyrinth zone when considering X-linked expression.

### Higher Expression Level Divergence at the Maternal–Fetal Interface

We next asked whether genes associated with nutrient transfer contribute disproportionally to interspecific expression level divergence in the mouse placenta. To this end, we performed a series of differential expression (DE) tests between placental tissues of *M.’m.’domesticus*, *M.’m.’musculus*, and *M. spretus* and tested for significant overrepresentation of DE genes associated with the labyrinth zone or more generally with nutrient transfer.

**Fig. 4.**
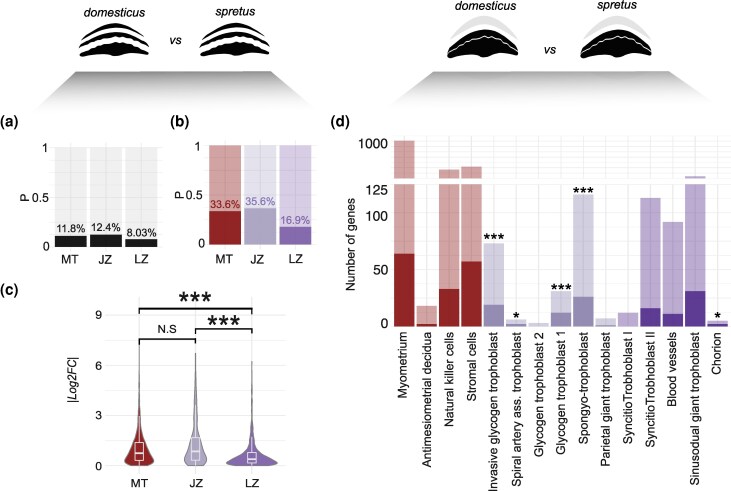
Interspecific differences in gene expression level divergence across placental layers. a to c) DE across placental layers between *M.’m.’domesticus* and *M. spretus*. a) Proportions of genes showing DE between species relative to the complete transcriptome of each placental layer. b) Proportions of layer-induced genes showing DE between species. c) Distribution of |*log2FC*| values across sets of genes with induced expression. Stars mark significance for a two-sided Wilcoxon ranked sum test in pairwise comparisons (*P < 0.05, ***P < 0.01). d) DE between *M.’m.’domesticus* and *M. spretus* using whole fetal placenta tissue and incorporating single-cell expression data to link DE genes to placental layers. Bar plots show the relative abundance of DE genes among genes associated with each cell-type. Stars mark significance of a test for overrepresentation of differentially expressed genes among cell-type-biased genes (*P < 0.05, **P < 0.01, ***P < 0.001).

First, we performed pairwise DE tests within each placental layer between *M. m domesticus* and *M. spretus*. When considering all genes expressed, the junctional zone showed proportionally more DE (1,408 DE genes; 12.4% of genes tested), closely followed by the maternal tissue (1,473 DE genes, 11.8%), and then the labyrinth zone (965 DE genes, 8.03%; Fig. 4a). When considering only layer-induced genes, the junctional zone showed the largest proportion of DE genes (62 genes, 35.6%), followed by the maternal tissue (512 genes, 33.6%), and the labyrinth zone (94 genes, 16.9%; Fig. 4b). The extent of expression level divergence among induced genes was also greater in the junctional zone (median |*log2FC*| = 0.736), closely followed by maternal tissue (median |*log2FC*| = 0.735), and the labyrinth zone (median |*log2FC*| = 0.473; Fig. 4c).

These results suggest increased expression level divergence in the junctional zone and maternal tissue compared to the labyrinth zone, a pattern seemingly at odds with the prediction of elevated divergence related to nutrient transfer. However, the junctional zone is the most compositionally complex layer and the most difficult to dissect cleanly, raising concerns that estimates of DE in this layer may be inflated by technical variation in cellular composition of the dissected layers.

To address this concern, we performed a second test of DE between *M.’m.’domesticus* and *M. spretus* using whole fetal placenta samples and single-cell expression data (Wu et al 2024) to associate DE with specific cell types. Although rare cell types can be underpowered for DE, this approach helps overcome technical limitations associated with layer-enriched dissections. Analysis of single-cell expression data yielded 2,518 genes associated with individual cell types, of which 1,710 were assigned maternal tissue cell types, 572 to cell types from the labyrinth zone, and 236 to cell types from the junctional zone. Of 1,367 total DE genes, 296 were associated with placental cell types. Five cell types’were significantly enriched for DE, four from the’junctional zone (Invasive Trophoblast, Glycogen Trophoblast, Spongiotrophoblast, and spiral artery trophoblast; Hypergeometric *P* < 0.05) and one from the Labyrinth zone (Chorion, Hypergeometric *P* < 0.05; Fig. 4d). Functional enrichment analysis in the set of all DE genes identified enrichment for cell-signaling and immunological pathways (e.g. Cytokine-cytokine receptor interaction, Neuroactive ligand-receptor interaction; Chemical carcinogenesis; Hypergeometric, *P* < 0.05) but not for pathways associated with nutrient transfer (Table S8, Supplementary material online). Thus, analysis from layer-enriched dissections and from whole fetal placenta both placed the maternal–fetal interface as the primary site of interspecific expression divergence.

Lastly, to identify DE genes showing recent, lineage-specific expression divergence, we performed all pairwise DE tests among *M.’m.’musculus*, *M. m domesticus*, and *M. spretus* from whole fetal placentas and used *M. spretus* as an outgroup to infer whether expression changes were derived on the *musculus* or *domesticus* lineage. We identified 871 DE genes between *M.’m.’musculus* and *M.’m.’domesticus*, of which 221 and 238 were consistent with derived expression level changes in *M.’m.’musculus* and *M.’m.’domesticus*, respectively (Fig. S9, Supplementary material online). These 459 genes also showed enrichment for junctional zone cell types (Glycogen Trophoblast and Spongiotrophoblast; hypergeometric *P* < 0.001) and were functionally enriched for immune-related terms (e.g. Complement and coagulation cascades, hypergeometric *P* = 0.008; Table S8, Supplementary material online), further supporting an association between the maternal–fetal interface and gene expression divergence between *Mus* species.

### Interspecific Divergence of Parent-of-origin Expression

**Fig. 5.**
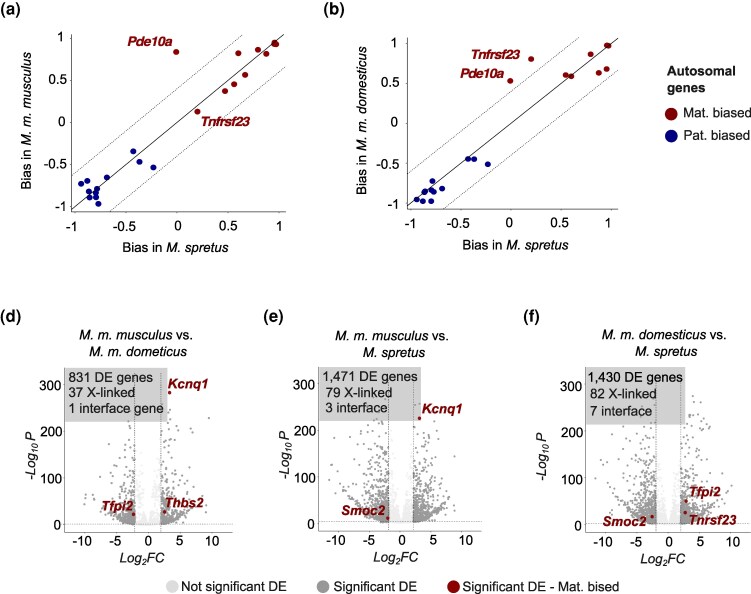
Evolutionary divergence in imprinted expression and gene expression levels of the *M. musculus* placental imprintome. a to c) Pairwise DE tests highlighting contributions of autosomal imprinted, X-linked, and interface genes to DE. d and e) Divergence in the extent of parent-of-origin expression bias in autosomal genes between each subspecies of *M. musculus* and *M. spretus*. Solid diagonal line indicates equal bias in the two lineages, and dotted diagonal lines delimit bias score differences greater than 0.4 between lineages. Plots show only genes with validated imprinted status and sufficient power for ASE in all crosses.

We next evaluated the contribution of imprinted genes to interspecific expression divergence. To do this, we first tested if imprinted genes contribute disproportionally to DE in the fetal placenta between species. Autosomal imprinted genes were not overrepresented among DE genes in any comparison (Hypergeometric > 0.05 in all comparisons, Fig. 5a-c). For example, of 871 DE genes between *M.’m.’musculus* and *M.’m.’domesticus* (6.9% of genes tested), only two genes (*Kcnq1*, *Tfpi2*; Fig. 3a) were imprinted (5.2% of imprinted genes tested). In the comparison between *M. domesticus* and *M. spretus*, 1,367 genes were DE (10.8% of genes tested), of which three were imprinted (*Smoc2*, *Tfpi2*, and *Tnsfr23*). Maternally biased, X-linked genes were marginally overrepresented among DE genes between *M.’m.’musculus* and *M. spretus* (57 genes, 12.7%; hypergeometric *P* = 0.04) and among DE genes between *M.’m.’domesticus* and *M. spretus* (64 genes, 13.3%; hypergeometric *P* = 0.015).

Next, we tested if imprinted genes showed evidence for turn-over of imprinted status between *M. spretus* and *M. musculus*. To do this, we calculated corrected parent-of-origin bias scores for *M. spretus* and compared parent-of-origin bias scores between the species. We limited our comparison to genes with validated imprinted status in our *M. musculus* model and with power to detect allele-specific expression in all crosses. Of 23 genes tested, only two showed variation in parent-of-origin bias across lineages: *Pde10a* showed biallelic expression in *M. spretus*, and *Tnfrsf23* showed weak maternally biased expression in *M.’m.’musculus* and *M. spretus* compared to *M.’m.’domesticus* (Fig. 5d and e), suggesting imprinted status of this gene might be restricted to the *M.’m.’domesticus* lineage. Overall, our results do not support a disproportionate contribution of imprinted genes to interspecific expression divergence and reveal considerable conservation of the placental imprinted landscape between *M. musculus* and *M. spretus*.

## Discussion

We combined a series of experimental mouse crosses, whole and layer-enriched placental transcriptomes, published single-cell expression data, and a novel analytical model for allele-specific bias to study the evolution of gene expression across functional layers and cells of the mouse placenta. Our results reveal important links between core elements of placental function and the evolution of gene expression between closely related species of mice, providing novel insights into how maternal–fetal conflict and other processes have shaped molecular evolution of the placenta. Below, we discuss the insights and limitations of our findings within the context of placental functional biology, molecular evolution, and mammalian diversity.

### Placental Tissue Specialization Shapes the Evolution of Placental Gene Expression

The eutherian placenta is distinctive for its diversity of cell types and degree of maternal–fetal tissue integration (Wagner et al. 2014; Stadtmauer et al. 2025). The intimate interaction of genetically distinct tissues and the evolutionary tensions that it might generate has inspired elegant and influential theoretical frameworks on the evolution of placental expression (Moore and Haig 1991; Haig 1993; Haig 1996). This theory has, in turn, motivated empirical research that has uncovered genetic signatures of parental conflict in the evolution of imprinted gene expression, such as reciprocal imprinting and co-expression of functionally antagonistic genes (DeChiara et al. 1991; Babak et al. 2015) and increased expression divergence at imprinted genes over deep evolutionary scales (e.g. mouse vs. humans; Babak et al. 2015). Nevertheless, many previous works have focused on genes imprinted across many tissues or developmental stages, even excluding aspects of placental imprinting due to the technical challenges of maternal contamination (Babak et al. 2015). Thus, the relative contribution of maternal–fetal conflict to placental expression evolution remains poorly understood (Patten et al. 2014), as does the role of distinct placental functions in shaping the landscape of imprinting and interspecific gene expression divergence more broadly.

Our study revealed two major patterns of placental expression that inform a more integrative perspective of how distinct elements of placental biology appear to influence molecular evolution within this critical tissue. First, we found a strong association between parent-of-origin expression and the labyrinth zone (Fig. 3a-b), consistent with a recent single-cell study that identified compartmentalization of paternally expressed genes across placental layers (Wu et al. 2024). We found that the labyrinth zone is enriched for both autosomal and X-linked genes (see below). Collectively, these results suggest that both maternal and paternal imprinting have been selected for specifically within the layer where physiological exchange between maternal and fetal blood occurs, a pattern that broadly supports the role of conflict over resource allocation as an important driver in the evolution of genomic imprinting (Moore and Haig 1991). Consistent with this model, several of the imprinted genes induced in the labyrinth zone have been linked to the regulation of angiogenesis and cell proliferation (e.g. *Peg10*, *Mest*, *Igf2*, *Dlk1*, *Rtl1*, *Plagl1*; DeChiara et al. 1991; Mayer et al. 2000; Constância et al. 2002; Sekita et al. 2008; Tunster et al. 2018; Xie et al. 2018; Starks et al. 2020) and likely influence the size and nutrient transfer capacity of this layer in the mature placenta.

Second, we found that patterns of interspecific divergence of gene expression also differed between placental layers. Maternal–fetal conflict has been generally assumed to fuel interspecific divergence of placental genes and pathways that in turn contribute to the rapid evolution of reproductive isolation between mammal species (Crespi and Nosil 2013). Therefore, we predicted that the labyrinth zone would also show elevated gene expression divergence between species, especially for imprinted genes thought to be directly involved in maternal–fetal conflict. Contrary to these predictions, we found that genes induced in the labyrinth zone showed lower divergence in gene expression levels between species (Fig. 4). We also found minimal variation in imprinting status and expression levels among imprinted genes (Fig. 5). In contrast, we found clear evidence for elevated expression divergence for genes more highly expressed in the junctional zone and maternal tissues, suggesting an important role for maternal–fetal interactions in driving interspecific molecular divergence.

Some caution is called for when interpreting these divergence results. The junctional zone is the most compositionally complex placental layer and thus may be sensitive to variance in cellular composition of layer dissections that could partially confound analyses of gene expression divergence between experimental groups (Good et al 2010, Hunnicutt et al 2022). Our follow-up analyses considering whole placenta transcriptome divergence in the context of single-cell expression data argued against technical artifacts; however, it is possible that some of the signal derives from evolved differences in overall placental cellular composition between these mouse lineages. If true, then our results would still point to elevated evolutionary divergence of the junctional zone, albeit through different mechanisms. Nonetheless, increased divergence at the junctional zone is also supported by previous studies that found increased protein sequence (Chuong et al. 2010) and DNA methylation (Decato et al. 2017) divergence in this layer relative to the labyrinth zone, highlighting the importance of this interface in the evolution of the rodent placenta.

### The Role of X Chromosome in the Evolution of Placental Gene Expression

Previous studies have shown X-linked enrichment for placental genes in mice (Khil et al. 2004) and hamsters (Moore et al. 2022). In parallel, the X chromosome also plays outsized roles in placental diseases in humans (Hemberger 2002; Del Gobbo et al. 2020) and disrupted placental development in multiple hybrid rodent crosses (Zechner et al. 1996; Hemberger et al. 1999; Vrana et al. 2000; Brekke et al. 2021). Our results further support the notion that the X chromosome plays a major role in the evolution of placental gene expression, revealing that enrichment for X-linked expression in the placenta is driven by genes expressed within the labyrinth zone (Fig. 3a and c).

The evolutionary drivers of these intriguing patterns are unclear. While population genetics theory predicts enrichment of X-linked expression in male and female reproductive tissues under some conditions (Vicoso and Charlesworth 2006), the biology of the rodent placenta may generate additional selective regimes that strongly favor X-linked expression. In general, hemizygosity of the X chromosome is predicted to result in faster fixation of mutations that benefit males because recessive alleles will be directly exposed to selection in males (Rice 1984). Given this, enrichment for X-linkage is generally predicted for male reproductive tissues in XY sex determination systems if beneficial mutations are on average recessive (Rice 1984; Charlesworth et al. 1987). Female bias may also emerge under some conditions, for example, if female-beneficial dominant mutations are common (Charlesworth et al. 1987). However, the rodent placenta is unusual among eutherian tissues in that XCI is not random but paternally imprinted, resulting in silencing of the paternal X chromosome (Takagi and Sasaki 1975). Thus, X-linked placental genes in rodents experience both maternal-biased transmission in populations and more efficient selection due to largely maternal (haploid) expression in both sexes.

In addition to general genetic considerations, enrichment for X-linked expression specifically within in the labyrinth zone could reflect genetic mechanisms that help resolve increased maternal–fetal conflict in this layer. If these patterns are an outcome of conflict, it is worth noting that the very large contribution of X-linked expression to imprinted expression assures an overwhelming bias toward maternal expression within the rodent placenta. That is, although maternally versus paternally imprinted genes appear to be equally represented on the autosomes, approximately 95% of all genes showing parent-of-origin expression are maternally biased once accounting for the X chromosome. Given this overwhelming maternal bias, it is possible that other evolutionary forces are at play. For example, enrichment for X-linked expression in rodent embryonic placental cells overall is also consistent with predictions of the mother-offspring coadaptation theory (Wolf and Hager 2006; Patten et al. 2014; Wolf and Brandvain 2014), which conceives maternal expression in the placenta as a mechanism to minimize negative epistatic interactions between maternal and fetal genes. However, under this model, we would have seemingly expected stronger enrichment at the maternal–fetal interface, where most endocrine interactions between maternal and fetal cells occur. Evaluation of layer- and cell-specific patterns of X-linked expression in other systems that do not show imprinted XCI could provide insights into its significance for placental biology.

### Maternal Tissue Contamination, Imprinting, and Gene Expression at the Maternal–Fetal Interface

The intricate integration of maternal and embryonic tissues characteristic of invasive placentation presents both evolutionary and methodological challenges. From an evolutionary perspective, deep trophoblast invasion requires finely tuned maternal immune tolerance to prevent embryonic rejection without compromising pathogen defense (Zeldovich and Bakardjiev 2012; Ander et al. 2019). Methodologically, this integration complicates allele-specific expression analyses due to the inevitable risk of contamination of embryonic and maternal placenta samples, which can confound the interpretation of imprinted expression signals (e.g. Proudhon and Bourc’his 2010). This technical limitation has generated uncertainty over the imprinted status of novel candidates showing maternally biased expression in the mouse placenta (Wang et al. 2011; Okae et al. 2012; Finn et al. 2014), with validation often requiring complex experimental procedures such as embryo transplants (Okae et al. 2012).

Our custom analytical model provided an effective computational approach to minimize the impact of maternal contamination on allele-specific expression of most genes expressed in the placenta. By largely eliminating genome-wide contamination signals (Fig. S6, Supplementary material online), our model enabled robust and reproducible identification of most known imprinted genes. Unexpectedly, this approach also uncovered 18 candidate genes with maternally biased expression, many of which share a spatial expression pattern strongly associated with the interface of trophoblast invasion (Fig. 2). The detection of these genes was fortuitous as their spatially restricted and cell-specific expression at the maternal–fetal interface violates a key assumption of our model, and would be likely to result in maternally biased expression regardless of their true imprinted status.

Although a confounding factor in the identification of imprinted genes per se, several lines of evidence suggest that this distinct subset of maternally biased genes is intimately linked to the biology of maternal–fetal interactions in the placenta. First, most of these genes encode receptors and ligands involved in immunomodulation and cell invasion (Table S7, Supplementary material online). Second, they exhibit co-expression between maternal and fetal cells (Fig. 2b). Third, some of these genes have been independently identified as markers of placental cell types associated with trophoblast invasion in mammals. A recent comparative analysis of placental cell-specific expression across major mammalian lineages identified a paralog of one of these interface genes (*Adamts5*) as a marker for novel trophoblast cell types in macaque and humans (both species with highly invasive placentation) and six of them (*Mfap5*, *Adamts5*, *Erv3*, *Tfpi2*, *Tnfrsf11b*, *Tnfrsf23*) as markers of a specialized endocrine decidual cell type thought to interact with embryonic trophoblast in mouse (Stadtmauer et al. 2025). *Erv3* has also been identified as maternally biased and shows disrupted expression in interspecific hybrids in hamsters (Brekke et al. 2016; Brekke et al. 2021). Taken together, these results highlight the importance of these genes in trophoblast invasiveness and interactions with maternal tissues, two hallmark features of mammalian placentation.

## Conclusions

Our comparative framework spanning three closely related mouse lineages and three specialized placental layers revealed key links between core elements of placental biology and the evolution of gene expression in this unique reproductive tissue. After correcting for maternal tissue contamination, we found that the labyrinth zone was the primary site of imprinted placental expression with significant enrichment of both autosomal and X-linked genes showing parent-of-origin expression. This finding suggests a strong functional connection between nutrient exchange and the evolution of both maternal and paternal placental imprinting, albeit with a predominance of maternally biased expression driven by the X chromosome. We also found substantial differences in the extent of interspecific divergence of gene expression levels between placental layers. However, unlike imprinting, the highest divergence was observed for genes with higher expression in the junctional zone and maternal tissues. Overall, autosomal imprinted genes showed patterns of allele-specific expression that were largely conserved among lineages. Collectively, these new insights into the role of tissue specialization and maternal–fetal interactions in the evolution of placental gene expression in rodents provide much-needed data on the evolution of the mammalian placental gene expression across shallow evolutionary scales.

## Methods

### Experimental Overview

We used a series of experimental crosses between strains and subspecies of house mice to study the functional specialization and evolution of placental expression. An overview of the experimental crosses, tissue sampling, and genomic data collection is provided in Fig. 1 and Fig. S1 (Supplementary material online). The rationale for our experimental design is outlined in the *Results*.

### Genetic Crosses and Tissue Collection

We used six wild-derived inbred strains sampled from two species of mice, *M. musculus* and *M. spretus*, spanning ∼1 to 3 million years to a most recent common ancestor (Galtier et al. 2004; Suzuki et al. 2004; Fabre et al. 2012). *Mus musculus* was represented by two wild-derived inbred strains of *M.’m.’musculus* (PWK/Phj and CZECHII/Eij, hereafter: mus^PWK^ and mus^CZII^) and two wild-derived inbred strains of *M.’m.’domesticus* (WSB/EiJ and LEWES/EiJ, hereafter: dom^WSB^ and dom^LEW^). *Mus spretus* was represented by two wild-derived strains (STF/Pas and SFM/M, hereafter: spret^STF^ and spret^SFM^) that have been maintained in a closed colony since establishment in 1982. All animals used in this study were bred at the University of Montana using mice derived from the Jackson Laboratory (*M. musculus*) or from the Montpellier Wild Mice Genetic Repository (*M. spretus*). Classic laboratory strains of *M. musculus* were not used in this study. All experimental procedures were performed in accordance with University of Montana Institutional Animal Care and Use Committee regulations (IACUC protocol #068-21).

We generated reciprocal crosses between strains within’each of the three lineages (mus^PWK^ x mus^CZII^, dom^WSB^ x dom^LEW^, spret^STF^ x spret^SFM^) and one additional intersubspecific cross between *M.’m.’musculus* and *M.’m.’domesticus* (mus^PWK^ x dom^WSB^) to increase our power to quantify allele-specific expression and DNA methylation (see Fig. S1 for sampling details, Supplementary material online). Since these wild-derived strains rarely have visible copulatory plugs, female mice were left with males until pregnancy was confirmed, weighed at pairing, at 14 d post-pairing, and every 2 d following. Weight gain was used in conjunction with visible cues such as nipple prominence and relative girth to determine onset and stage of pregnancy, determined for each strain through similar measurements in breeding mice taken during colony maintenance. Females were sacrificed in late-stage gestation and embryonic development was determined via Theiler stage (TS). We only kept samples from conceptuses staged between TS24 and TS26 (Theiler 1989).

We performed two types of placenta dissections from these crosses. For whole embryonic placenta dissections, maternal tissue (including decidua and the attached myometrium) was separated from embryonic portion of the placenta (Fig. 1b, left), and each embryo and embryonic placenta was weighed. Embryonic placental samples were immediately sectioned into four even quadrants along the sagittal and transverse axes (i.e. preserving the layer architecture within each section). For layer-enriched dissections (Fig. 1b, right), the maternal tissue was separated from embryonic tissues, then the junctional zone was carefully separated from the labyrinth zone (Qu et al. 2014), and all layers were snap-frozen in liquid nitrogen. Each pup and its corresponding placenta were sexed through embryo genotyping following Tunster (2018). We selected 32 layer-enriched dissection samples (8 maternal tissue, 12 junctional zone, and 12 labyrinth zone samples) from two cross types (dom^WSB^ x dom^LEW^, spret^SFM^ x spret^STF^) for RNA-seq. For each of the four reciprocal crosses, we selected 20 whole embryonic placenta samples (five per sex per cross direction, *n* = 80 total) and eight corresponding maternal tissues (2 per sex per cross direction, *n* = 32 total) for RNA-seq. From this larger RNA experiment, we chose four samples per cross direction (two per sex) from a reciprocal (mus^PWK^ x dom^WSB^) cross for whole genome bisulfite sequencing (WGBS, *n* = 8 total).

### Library Preparation

Placental tissue was homogenized in TriReagent RT (Molecular Research Center, cat. no. RT111) using a Qiagen TissueLyser (cat. no. 85300), and RNA was extracted using a hybrid TriReagent—RNeasy spin column method. Following TriReagent phase separation, the aqueous phase was used as input to a RNeasy Mini column (Qiagen, cat. no. 74106), after which the manufacturer's protocol was followed. Once all tissue collections were complete, we prepared RNA-seq sequencing libraries in blocks containing an equal representation of all cross-types to minimize batch effects. Libraries were generated using the Kapa mRNA HyperPrep kit (Roche, cat. no. KK8581), barcoded with unique dual index adaptors (Roche, cat. no. KK8727), and pooled across two lanes of 150 bp PE Illumina NovaSeqS4 (Novogene, Sacramento, CA). For samples also prepared for WGBS, high-quality DNA was extracted from flash-frozen placental tissue using a DNeasy Blood and Tissue Kit (Qiagen, cat. no. 69506), and bisulfite converted with the Zymo EZ DNA Methylation Gold spin column kit (cat. no. D5005). Libraries were prepared with the transposase-based Accel-NGS Methyl-Seq DNA Library kit (Swift Biosciences, cat. no. 30096) and barcoded with the Methyl-seq set A indexing kit (Swift Biosciences, cat. no. 36024), following manufacturer's instructions. WGBS sequencing was performed on a single lane of 150 bp PE Illumina NovaSeqS4 and sequenced by Novogene (Sacramento, CA).

### Transcriptome Data Processing

RNA-seq reads were filtered and trimmed using *Trimmomatic* (Bolger et al. 2014), with the following settings [ILLUMINACLIP:TruSeq3-PE.fa:2:30:10 LEADING:3 TRAILING:3 SLIDINGWINDOW:5:20 MINLEN = 36]. To reduce reference bias, strain-specific pseudo-genomes were downloaded from the Mouse Collaborative Cross database (WSB, LEWES) or generated with *Modtools* (Huang et al. 2014) using resequencing data mapped to the *M. musculus* (PWK, CZII) or *M. spretus* (STF, SFM) reference genome assemblies (GRCm38 and SPRET_EiJ_v1, respectively). Trimmed RNA-seq reads from each sample were mapped to parental pseudogenomes using *Hisat2* (Kim et al. 2019). A custom python script was used to reformat *Hisat2* alignment files to allow compatibility with the *ModTools* pipeline (https://github.com/LF-Rodriguez/Mouse_imprinting_evolution). Genome mapping coordinates were then converted to a common reference coordinate system and merged into a single alignment file using *Lapels* (Huang et al. 2014). All reads were assigned maternal, paternal, or undetermined origin based on differences in mapping quality to each parental pseudogenome using *Suspenders* (Huang et al. 2014). Two types of expression matrices were generated. First, all alignments from each sample (regardless of their parent-of-origin) were used as input to *featureCounts* (Liao et al. 2014) to generate gene expression matrices. Then, reads unambiguously assigned to each parental strain were grouped into separate alignment files and used as input to *featureCounts* to generate allelic expression matrices.

### Layer-induced and Differential Expression Analyses

We used 36 layer-enriched samples (six from each layer for each species) to detect layer-induced expression. We first categorized genes as either expressed or not expressed in each layer and then identified genes with’expression enriched for a particular layer (i.e. layer-induced expression) following Kopania et al. (2022) and Larson et al. (2016). Genes were considered “expressed” in a layer if they showed a z-FPKM normalized expression level > −3, following Hart et al. (2013). A gene was considered “induced” if its median expression in the focal layer was at least 2 × greater than the sum of its median expression in the other two layers. Similarly, a continuous layer-specificity score was also calculated to evaluate threshold effects on differences in induced expression between species. This score was calculated as the difference between the median expression of a gene in the focal layer and the sum of its median expression in the other two layers.

### Analysis of Single-cell Data

Two publicly available datasets (Jain and Tuteja 2021; Wu et al. 2024) were used to validate our layer-enriched dissection approach and to evaluate spatial patterns of’gene expression of genes of interest. For layer-induced expression, we tested for overrepresentation of placental cell type markers using the online tool’PlacentaCellEnrich (https://placentacellenrich.gdcb.iastate.edu). Additionally, we compared cell-type specificity scores across layer-induced gene-sets. These scores were calculated as the log2fold change values of DE tests between each cell type and all other placental cell types using Wu et al. (2024) single cell data-set and the cirrocumulus software (Li et al. 2020). For analysis of spatial gene expression patterns, we generated gene expression plots across placental layers from mature placenta samples (E14.5) from Wu et al. (2024) and the cirrocumulus graphical interface. We considered genes associated with the interface of invasion if they had a *P*-value < 0.05 and a log2FC > 2 in the invasive trophoblast versus all other cell types.

### Functional Enrichment Analysis

Functional enrichment analyses were performed using the *gost* function of the R package gprofiler2 v 0.2.1 (Raudvere et al. 2019; Kolberg et al. 2020). This package uses hypergeometric tests for enrichment of categories from GO, KEGG, REACTOME, MIRNA, and other databases within the subset of query genes, from which we excluded GO inferred from electronic annotations (automated annotations that have not been manually reviewed). For tests of overrepresentation of imprinted genes, we used all genes with power for allele-specific expression in placental tissue as background. For other tests, we used all genes expressed in the placenta as the background. In all cases, we used Bonferroni correction for multiple testing.

### Detection of Parent-of-origin Expression

We used whole embryonic placenta samples from reciprocal crosses to test for parent-of-origin expression bias. First, we filtered allele-specific expression matrices by removing genes without sufficient power for allele-specific expression (ASE) analysis in each cross type. For this, we required a minimum coverage of 20 reads over at least two diagnostic variants separated by at least 150 bp (read length) in at least four biological replicates. Next, we tested for parent-of-origin expression bias at each gene using a *χ*^2^ test on the contingency table composed of TPM-normalized read counts of each allele in each direction of the cross at a *P*-value cutoff of 0.01 after Bonferroni correction. Additionally, we quantified the extent of parent-of-origin bias using the bias score proposed by Wang et al. (2011) as follows. First, the variables P1 and P2 were defined as the proportion of gene expression originating from a reference allele when inherited from the mother (P1), and the proportion of gene expression of the same allele when inherited from the father (P2) (e.g. in a dom x dom cross, P1 = % WSB allele expression in a ♀dom^WSB^ x ♂dom^LEW^ cross, and P2 = % WSB allele expression in a ♀dom^LEW^ x ♂dom^WSB^ cross). Bias scores were calculated as the difference between P1 and P2, which ranges from −1 to 1, reflecting complete paternal and maternal bias, respectively. A gene was considered biased if its absolute bias score was equal to or greater than 0.4, corresponding to 70% or more expression originating from the maternally or the paternally inherited allele. This cutoff is the same as that used by Brekke et al. (2016) and slightly more conservative relative to other studies assessing parent-of-origin expression bias in mouse placentas (e.g. 65%, Wang et al. 2011; Andergassen et al. 2017).

### Estimation and Correction of Maternal Contamination

To try to account for the effect of maternal contamination in our dataset, we developed an analytical model akin to the model of Finn et al (2014). Generally, both models accounted for maternal contamination in two steps. First, the extent of maternal contamination across samples was calculated from deviations of a priori expectations of allelic imbalance. Second, the contribution from maternal contamination to parent-of-origin bias estimates was calculated for each gene as the product of the calculated sample contamination and a gene's maternal to embryonic placenta expression ratio (M:E).

Our model differs from Finn et al (2014) in two ways. First, our model did not rely on allelic balance expectations from known paternally expressed genes or from complete transcriptomes to estimate sample contamination. Instead, our model identified genes whose predicted allelic expression ratio was 1:1 (i.e. no allelic imbalance). Second, our model generated estimates of maternal to embryonic expression ratios for each gene specific to the maternal genotype for each cross. Estimates of maternal contamination were then used to calculate adjusted parent-of-origin bias scores by subtracting the predicted contribution of maternal contamination to observed scores on each gene. A detailed explanation of the model is provided in the Supplementary Methods.

### Calling Imprinted Expression

We used a hierarchical approach to identify genes with imprinted expression in the *M. musculus* placenta. First, we applied the model above to three different reciprocal cross-types within *M. musculus* (i.e. reciprocal dom x dom, mus x mus, mus x dom) to identify a robust set of genes showing parent-of-origin expression (see Fig. S5, Supplementary material online). Second, we excluded genes with a high maternal to embryonic expression ratio (M:E > 3) as these showed the greatest variation in expression levels. After these two corrections for maternal contamination, genes with a *χ*^2^ corrected *P*-value below 0.01 and a parent-of-origin bias score greater than 0.4 or less than −0.4 were considered maternally or paternally biased in each of the three cross types, respectively (Wang et al. 2011; Brekke et al. 2016). We then excluded genes that were not independently detected as imprinted in at least two of the three *M. musculus* cross types.

Next, we cross-referenced our corrected and replicated gene set with a reference list of genes whose imprinted status had been validated through either embryo transplant experiments, trophoblast cell cultures, or experimental manipulation of epigenetic marks (Table S9, Supplementary material online). We compiled this list using records from the Otago Imprinted Gene Catalog (https://corpapp.otago.ac.nz/gene-catalogue), the Geneimprint database (https://www.geneimprint.com), and Andergassen et al. (2017). Validated and paternally biased genes were classified as imprinted for subsequent analyses. All remaining maternally biased genes were excluded if they did not meet any of the following criteria: (i) a strong bias score (>0.7) under the most stringent threshold of contamination (maximum instead of median), (ii) not known to be highly expressed in blood relative to other tissues. To identify genes with high expression in blood, we combined standardized expression values from the bloodExpress database and the mouse ENCODE transcriptome dataset (https://www.encodeproject.org/awards/U54HG007004/). We considered a gene highly expressed in blood if it showed a blood expression level higher than the maximum of all other tissues. Maternally biased genes that passed these filters were kept in our final placental imprintome but treated as tentative candidates for imprinted expression (Fig. S5, Supplementary material online).

### DNA-methylation Analysis

DNA methylation analysis was performed using the *Methpipe* pipeline (Song et al. 2013). Filtered reads were mapped to the reference genome using *abysmal* (de Sena Brandine and Smith 2021), PCR duplicates were removed using the *duplicate-remover* script, and methylation levels were estimated at individual CpG sites using the *methcounts* and *symmetric-cpgs* scripts. The final CpG methylation calls were calculated after merging biological replicates to increase coverage. CpG sites covered by less than 10 reads were filtered out as suggested in the *Methpipe* manual. Filtered CpG methylation calls were used to detect hypomethylated regions using the *hmr* script and differentially methylated regions using the *dmr* script with default parameters.

For allele-specific methylation analysis, reads from each sample were separated by parent-of-origin based on informative sites and accounting for sequence changes induced by bisulfite treatment using a custom python script, *K-Padre* (https://github.com/LF-Rodriguez/k-Padre). To increase our power for detection of allele-specific hypomethylated and differentially methylated regions, maternal and paternal CpG methylation calls from all samples within a cross type were merged into single files using the *merge-methcounts* script (i.e. CpG methylation calls from PWK reads from all ♀mus^PWK^ x ♂dom^WSB^ samples and WSB reads from all ♀dom^WSB^ x ♂mus^PWK^ samples were merged to generate compiled maternal methylation calls). DMRs between the maternal and paternal genomes in the mus x dom reciprocal cross were considered parent-of-origin DMRs. These regions were considered physically linked to a gene if they were located within a gene body or within 2,000 bp of the gene boundaries. Parent-of-origin DMRs were considered linked to an imprinted cluster if they were physically linked to a gene within a known imprinted gene cluster.

### RNA in Situ Hybridization

Three mature whole placentas from *mus^PWK^ x mus^PWK^* crosses were harvested and preserved on optimal cutting temperature (OCT) solution, carefully frozen by submerging them in an ethanol bed resting on dry ice. Mounted tissue was cryosectioned at 10 µm and kept at −80 °C for immunohistochemistry. Samples were fixed with 37% paraformaldehyde at room temperature, then permeabilized using 70% ethanol for 1 h.’Probes were designed and ordered through Integrated DNA Technologies (IDT), adding FLAP sequences conjugated to Cy5 and Cy3. A complete list of probe sequences is provided in Table S10, (Supplementary material online). Probes were hybridized to target RNA in 10× NEB 3 buffer using a thermocycler with the following conditions: one cycle at 85 °C for 3 min, one cycle at 65 °C for 3 min, and one cycle at 25 °C for 5 min following the *Stellaris RNA FISH protocol for frozen tissue* but replacing FISH probes with FLAP-hybridized probes (Table S10, Supplementary material online).

### Differential Expression

Differential gene expression analyses were performed with the R package *DESeq2* (Love et al. 2014). First, gene expression values were standardized using zFPKM using species-specific transcript length estimates to filter lowly expressed genes. For every pairwise comparison, genes were kept for DE analysis if they were expressed (zFPKM > −3) expressed in at least 80% of biological replicates. Then raw counts were incorporated into the DESeq2 pipeline for DE analysis. Genes were considered differentially expressed if they showed an absolute value of *log2FoldChange* (*|log2FC|*) of at least one and a maximum adjusted *P*-value of 0.05.

One potential concern for the use of the DESeq2 pipeline is that interspecific differences transcript structure could potentially confound DE analysis. . To explore this issue, we estimated transcript lengths for *Mus* species combining existing gene models (i.e. reference gene annotations) and empirical transcript-length estimates derived from RNA-seq data. First, we obtained exon coordinates from annotations of *M.’m.’musculus*, *M.’m.’domesticus* and *Mus spretus*. Then, we used species-specific RNA-seq reads mapped to their correspondent reference genomes and re-calculated the length of annotated transcripts using RNA-seq contigs overlapping annotated exons (Fig. S10a-b, Supplementary material online). Differentially expressed genes were excluded post hoc if apparent expression differences could be attributed to differences in transcript length (Fig. S10 c-d).To identify lineage-specific shifts in expression levels, we combined the’intraspecific DE test (*M.’m.’musculus* vs. *M.’m.’domesticus*) with interspecific comparisons to *M.’spretus*, which we used as an outgroup. Expression levels of genes showing DE between subspecies were considered derived in the *M.’m.’musculus* lineage if they also were DE between *M.’m.’musculus* and *M. spretus* but not between *M.’m.’domesticus* and *M. spretus*. Conversely, expression levels of genes showing DE between *M.’m.’domesticus* and *M. spretus*, but not between *M.’m.’musculus* and *M. spretus*, were considered derived in the *M.’m.’domesticus* lineage. Genes that showed DE between subspecies but not between species, were not polarized as these genes showed a gradient of expression with the outgroup at an intermediate level.

### Evolutionary Divergence in Parent-of-origin Biased Expression

To study divergence of imprinted expression, we first calculated parent-of-origin expression bias corrected for maternal contamination using whole embryonic placenta samples from reciprocal *M. spretus* crosses. We then performed pairwise comparisons between each of the two *M. musculus* lineages and *M. spretus* (i.e. dom x dom vs. spret x spret; mus x mus vs. spret x spret) using genes identified as imprinted in *M. musculus* and with sufficient power to call allele-specific expression in the three cross types. To detect changes in imprinted status, we quantified differences in the strength of parent-of-origin expression between lineages. We considered a gene divergent if the difference in score bias was greater than 0.4 between lineages or if it showed nonsignificant parent-of-origin bias in one of’the lineages being compared. Given the replication required in our imprinting pipeline (Fig. S5, Supplementary material online), this contrast does not allow detection of gain of imprinting in *M. spretus*.

## Supplementary Material

evag120_Supplementary_Data

## Data Availability

All raw sequencing reads are archived in the SRA (bioproject PRJNA1020824). Accession numbers for individual libraries are provided in Table S11 (Supplementary material online). All expression data necessary to reproduce this study were deposited in figshare.com (https://doi.org/10.6084/m9.figshare.31985367).
