# Supplementary material for: Evolution of Gene Expression Across Functional Regions of the Mouse Placenta
Source: Genome Biol Evol. 2026 May 23;18(5):evag120. doi: 10.1093/gbe/evag120 (PMC13202214; doi:10.1093/gbe/evag120)
Supplement: evag120_Supplementary_Data [file evag120_supplementary_data.zip › GBE_Imprinting_evolution_Supplementary_figures_20260403.docx]

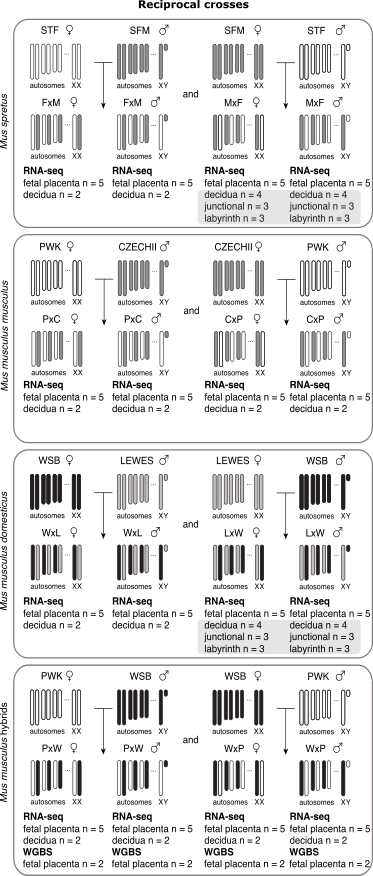


**Fig. S1.** Experimental crosses and sampling of genomic data. Design of reciprocal crosses from three *Mus* lineages used in this study, indicating specific wild derived inbred lines (PWK, CZECHII, WSB, LEWES, STF, and SFM) with sample sizes for each data type, mRNA sequencing (RNA-seq) and whole genome bisulfite sequencing (WGBS).


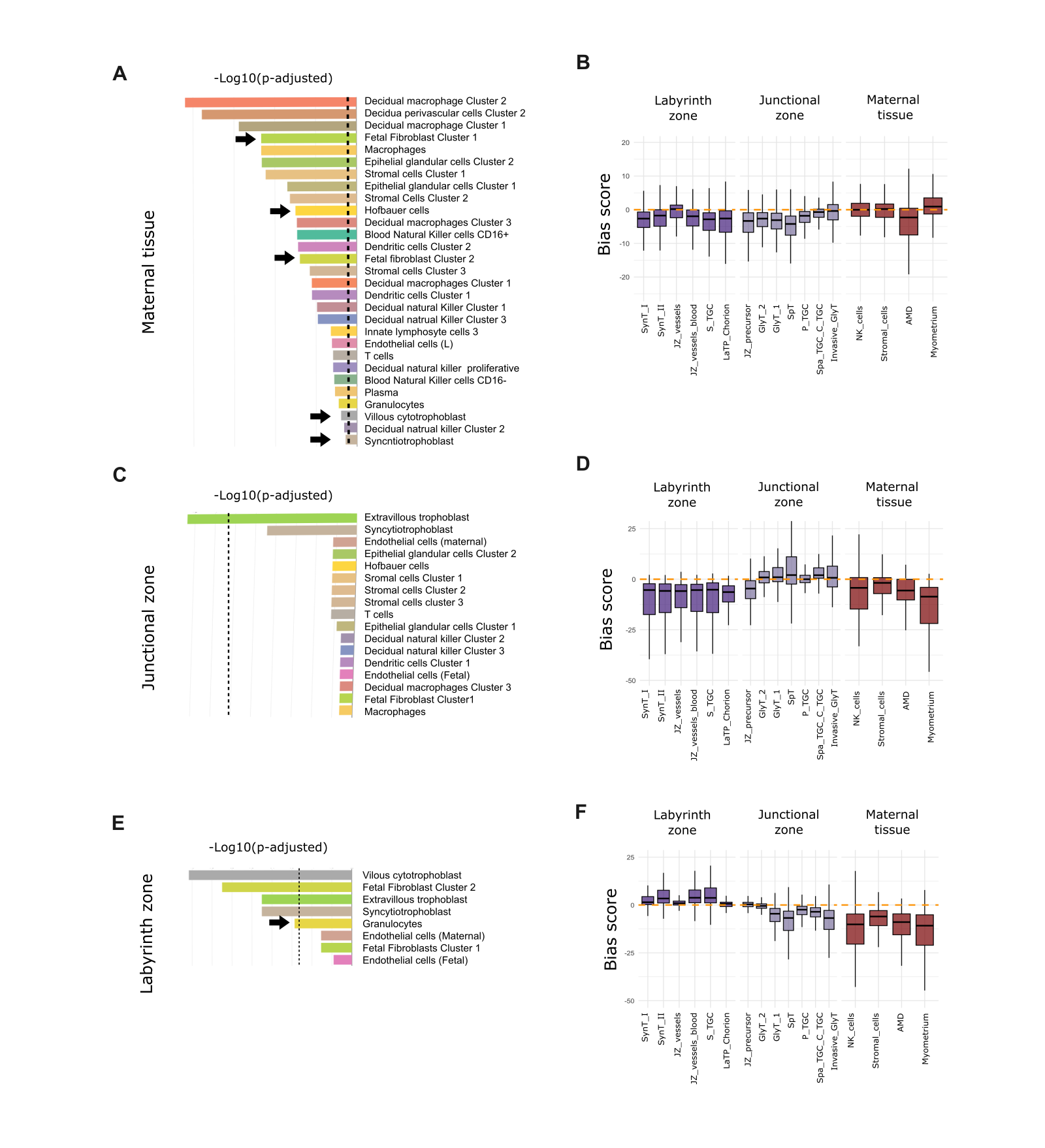


**Fig. S2.** Evaluation of layer induced expression. Sets of genes identified as layer-induced in our study were compared to two publicly available datasets: placentalCellEnrich (Jain and Tuteja 2021; left) and the spatio-temporal transcriptomic atlas of mouse placentation (Wu et al. 2024; right). (**A,C,E)** Results of enrichment (hypergeometric test) for placental cell-type markers among gene sets of induced expression. Bars show statistical significance for each cell type in the reference database. Dotted line marks a significance p-value threshold of 0.05 after correction for multiple testing. Arrows mark enrichment of markers from cell types outside the target layer. **(** **B,D,F)** Distribution of cell-type specific bias scores in reference data set (Wu et al., 2024) for each group of layer-induced genes in our study. Dotted line marks a bias score of zero. Positive scores reflect biased expression on each cell type.


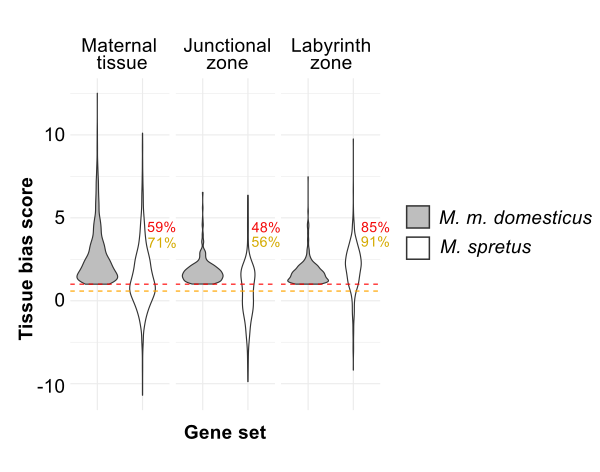


**Fig. S3**. Evaluation of threshold effects on the overlap of layer-induced expression between *M. m. domesticus* and *M. spretus*. Violin plot shows the distributions of quantitative measures of induced expression in *M. m. domesticus* and *M. spretus* using the set of layer-induced genes identified on *M. m. domesticus* as a reference. Induced expression scores were calculated as the difference between standardized median expression values (TPM) of a gene in the target layer, and the sum of its median expression in the other two layers. Red and orange dotted lines mark an induced expression score thresholds of 2 and 1.5 respectively. Percentages reflect the proportion of genes that show the same induction in *M. spretus* compared to *M. m. domesticus* under each threshold.


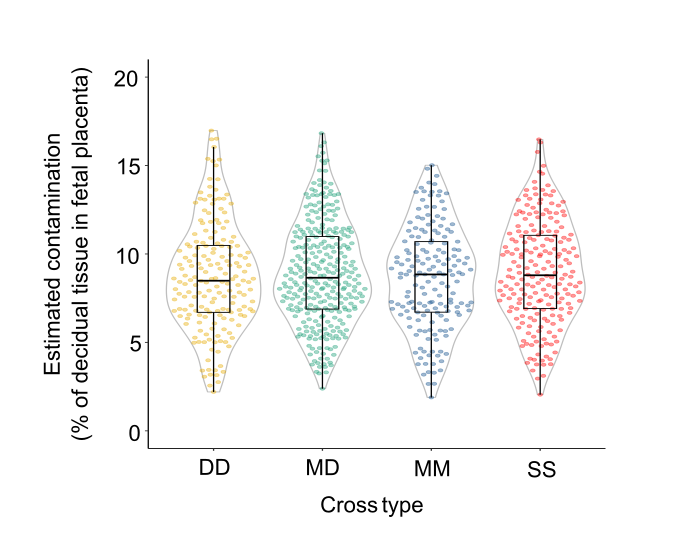


**Fig. S4.** Estimates of maternal contamination across cross types. Box plots show the distribution of estimates of maternal contamination calculated as the percent of decidual expression required to explain contamination in the fetal placenta. Each box plot displays estimates generated from diagnostic gene sets from the four cross types in this study. Cross types are coded as: DD: *dom* x *dom*, MD: *mus* x *dom*, DD: *dom* x *dom*, and SS: *spret* x *spret*.


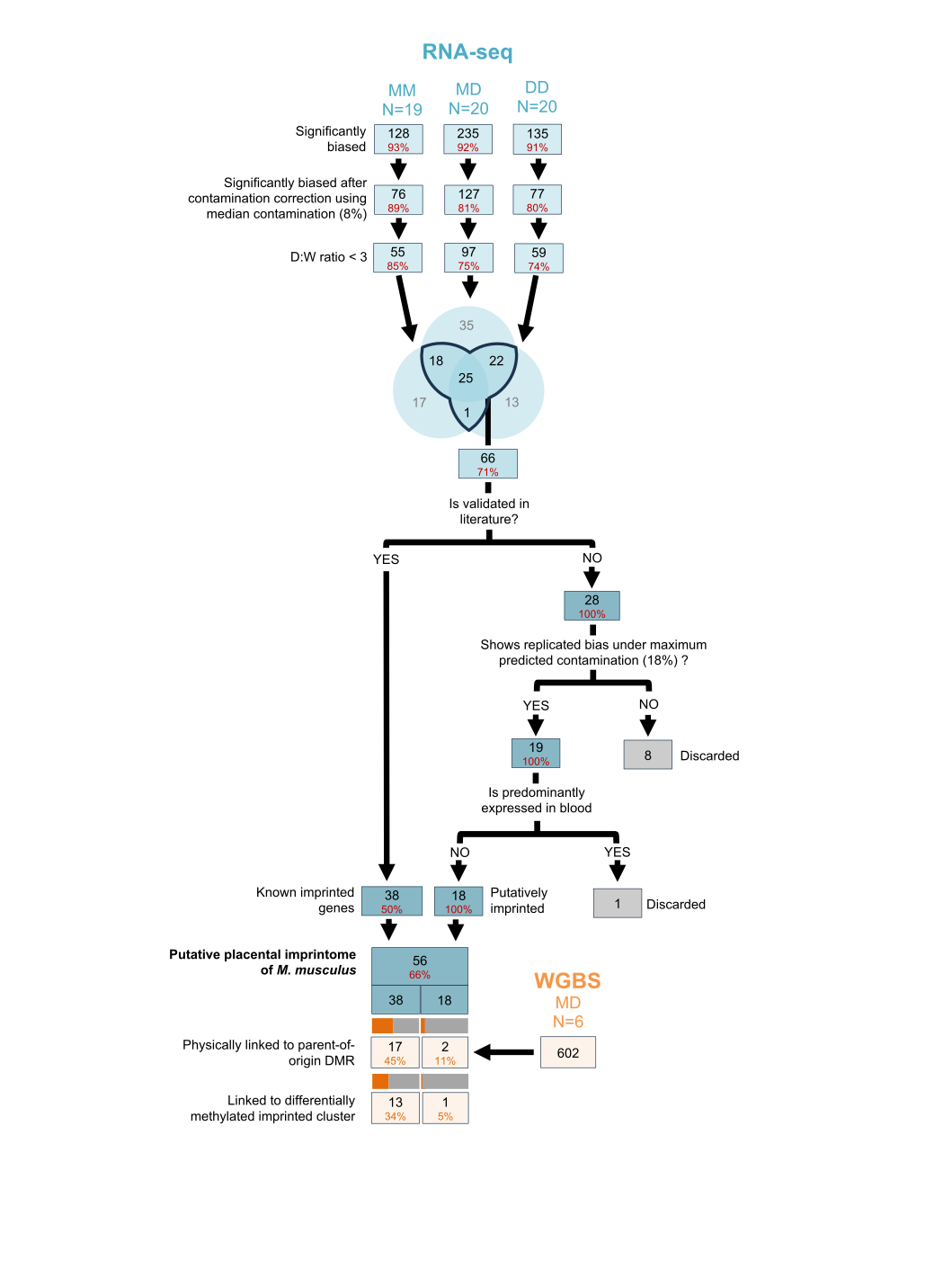


**Fig. S5.** Schematic representation of our pipeline for the identification of imprinted genes in the *M. musculus* placenta. Three independent cross types (each represented by 20 samples) using four wild-derived inbred lines of *M. musculus* were used to perform a replicated screen for imprinted expression using RNA-seq data. The resulting set of genes showing parent-of-origin expression bias was filtered on each cross using model-based correction, and strict thresholds to discard maternal contamination. Genes that showed parent-of-origin bias after correction in at least two crosses were considered candidates for imprinted expression. A whole-genome screen for parent-of-origin differentially methylated regions (DMRs) was performed independently and compared to results of RNA-seq. These analyses yielded 602 DMRs linked to genes expressed in the placenta, physically linked to 19 previously validated imprinted genes and three of the 18 candidates identified.


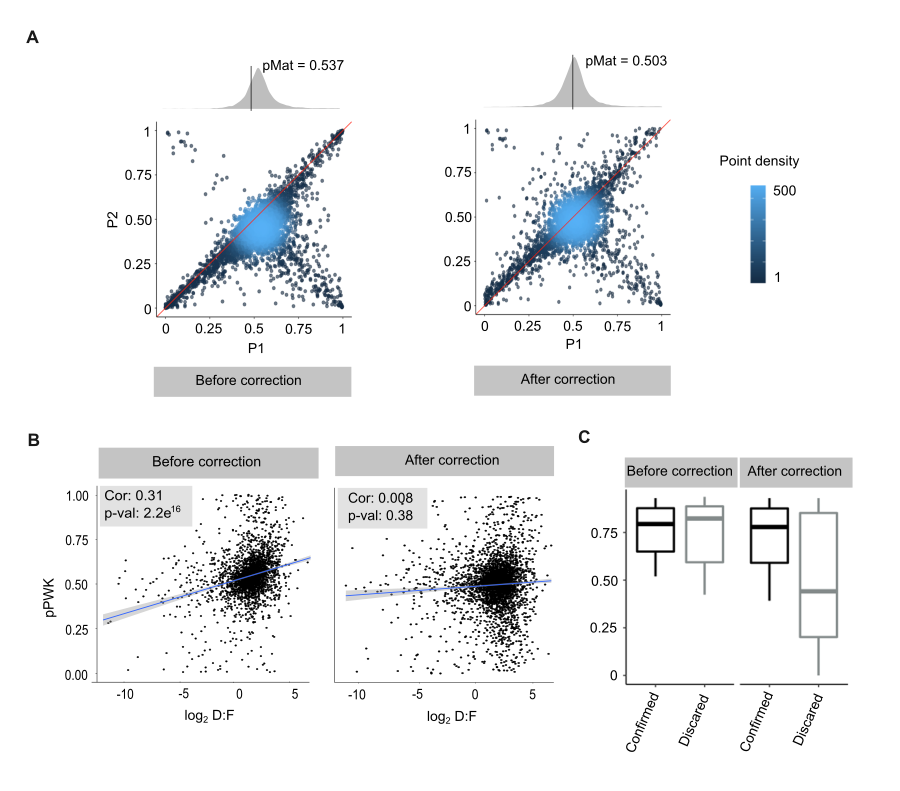


**Fig. S6.** Evaluation of model-based correction for maternal contamination in fetal placenta samples. (**A)** Genome wide distributions of P1, P2, and pMat values from *mus^PWK^* x *dom^WSB^* samples estimated before and after correcting for maternal contamination. (**B)** Correlation between expression maternal allele and relative expression in maternal cells across autosomal genes in the fetal placenta from ♀ *mus^PWK^* x ♂ *dom^WSB^* crosses before and after correction (**C)** Box plots showing the distribution of maternal bias scores of 19 genes scrutinized for contamination in another study (Okae et. al 2012) before and after correction. Genes are grouped in two categories: black box corresponds to eight genes with confirmed imprinted expression and grey box contains eleven genes detected as false positives in Okae et al (2012).


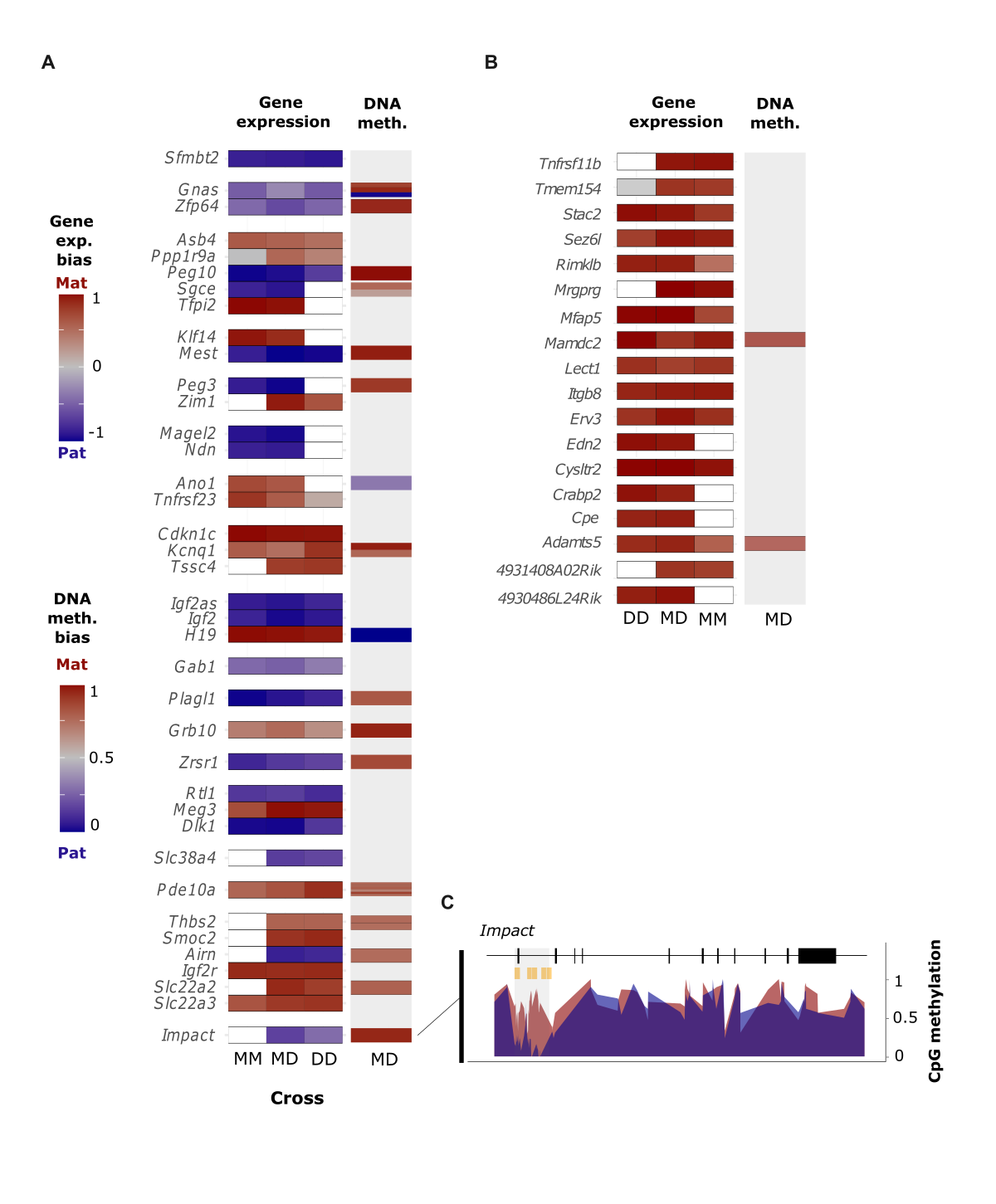


**Fig. S7.** Parent-of-origin expression and DNA methylation in the house mouse placenta. Patterns of parent-of-origin expression and DNA methylation in *M. musculus* at genes with validated imprinting status (A) and novel candidates (B). Genes are grouped by known imprinting clusters of the mouse genome. Individual tiles show average parent-of-origin bias scores for gene expression (P1 – P2 values) and DNA methylation (direction of bias for parent-of-origin DMRs) for three cross types of *M. musculus* wild-derived strains (DD: *dom* x *dom*, MD: *mus* x *dom*, MM: *mus* x *mus*). White tiles represent loci without sufficient power for allele-specific expression analysis in the cross. Note that a gene may display multiple DMRs overlapping its gene boundaries (*e.g*., *Gnas, Pde10a*). (C) Local allele specific DNA methylation at the known imprinted control region, *Impact*. Shown are exons (black), ENCODE annotated regions (yellow), CpG methylation of maternal (red) and paternal (blue) alleles, and DMRs boundaries (light gray).
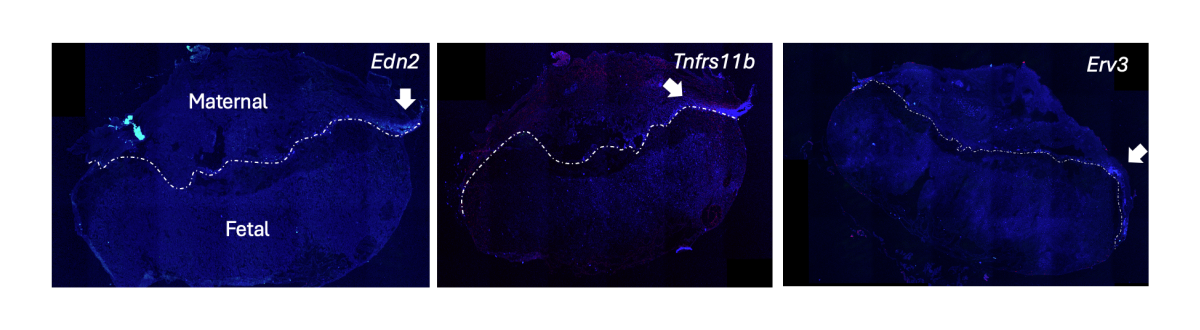


**Fig. S8.** Evaluation of spatial expression patterns of three candidate genes for imprinted expression in the mouse placenta. Pictures show transcript abundance of three genes using *in situ* hybridization. White dotted line shows the maternal-fetal interface in the cross section.

**
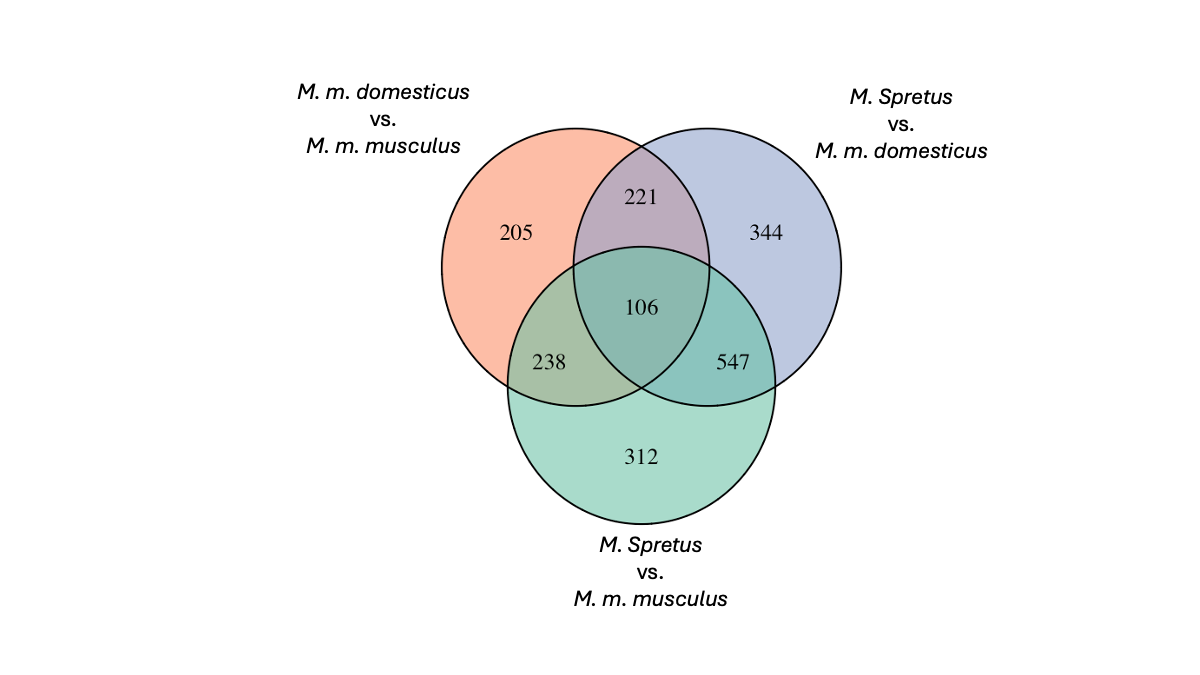
**

**Fig. S9** Overlap of DE gene-sets from all-pairwise DE tests among *Mus* lineages using whole fetal placenta samples.


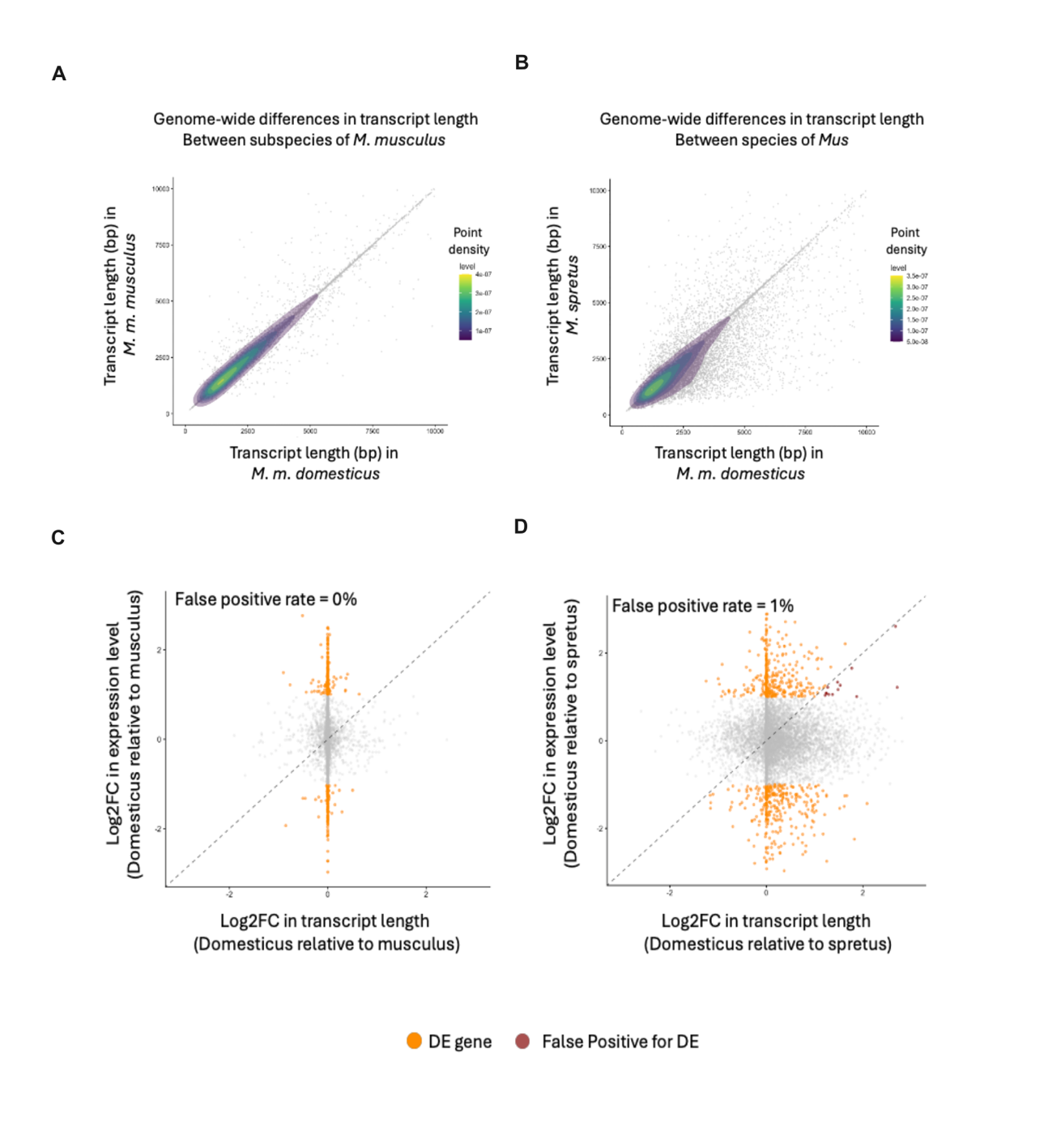


**Fig. S10.** Analysis of transcript length differences among *Mus* species / subspecies and impacts on differential expression. (A-B) Comparison of transcript length between subspecies of *M. musculus* (A) and between *M. m. domesticus* and *M. spretus* (B). (C-D) Impacts of transcript length difference in differential expression test. Colored dots correspond to genes identified as DE. Genes whose Log2Fold expression can be explained by a log2FC in transcript size, are considered putative false positives.


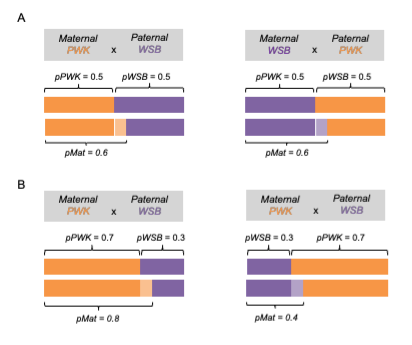


**Fig. S11.** Contamination on genes without allelic imbalance results in equal estimates of pMat in both directions of a reciprocal. The schemes show theoretical allele frequencies with and without contamination in both directions of a reciprocal cross (♀mus^PWK^ x ♂mus^WSB^ and ♀mus^WSB^ x ♂mus^PWK^) in a gene without allelic imbalance (A) and one with allelic imbalance (B). Solid bars represent expression of each of the two alleles in embryonic tissues and light color bars represent the effect of contamination on estimates of allelic expression ratios assuming a +0.1 shift of the maternal allele expression ratio induced by contamination. A) When a gene has no allelic imbalance (e.g., pPWK=0.5, pWSB=0.5), a shift of +0.1 in maternal allelic expression results in the same value of pMat (0.6) in both directions of the cross B) In contrast, when a gene shows allelic imbalance (e.g., pPWK=0.7, pWSB=0.3) estimates of pMat differ between the two directions of the cross due to the asymmetry in the true expression ratio of the maternal allele.
