# Supplementary material for: Evolution of Gene Expression Across Functional Regions of the Mouse Placenta
Source: Genome Biol Evol. 2026 May 23;18(5):evag120. doi: 10.1093/gbe/evag120 (PMC13202214; doi:10.1093/gbe/evag120)
Supplement: evag120_Supplementary_Data [file evag120_supplementary_data.zip › GBE_imprinting_evolution_Supplementary_methods_20260403.docx]

**Evolution of gene expression across functional regions of the mouse placenta**

*Fernando Rodriguez-Caro, Emily C. Moore, Ashlin Slanger, Jeffrey M. Good*

**Model based correction of maternal contamination**

Maternal and embryonic tissues are tightly integrated in the mouse placenta resulting in inevitable

contributions of maternal cells to estimates of gene expression in dissected fetal placenta samples (Proudhon and Bourc’his 2010). Maternal contamination in the mouse placenta has been modeled in the past (Finn et al. 2014), however, contamination estimates have varied dramatically depending on the set of diagnostic genes used. We used to measure maternal contamination using a modification of that implemented by Finn et al. (2014), aimed to increase the accuracy of contamination estimates and reduce false positives for parent-of-origin bias.

*Model of allelic expression in fetal placenta samples.* We assume that maternal contamination originates largely from remnant decidual cells in dissected fetal placenta samples. Thus, we modeled gene expression in the fetal placenta (*exp_Total_*) as the sum of transcripts originating from the maternally inherited allele (exp_Mat_), the paternally inherited allele (*exp_Pat_*) and from maternal alleles in contaminating decidual cells (*exp_Cont_*)(equation 1).

𝑒𝑥𝑝_𝑇𝑜𝑡𝑎𝑙_ = 𝑒𝑥𝑝_𝑀𝑎𝑡_ + 𝑒𝑥𝑝_𝑃𝑎𝑡_ + 𝑒𝑥𝑝_𝐶𝑜𝑛𝑡_

Because inbred genetic lines were used for experimental mice crosses, dams are expected to be

homozygous for most loci in the genome, thus, maternal transcripts from decidual cells cannot be

differentiated from transcripts originating from the maternally inherited allele in fetal placenta cells using sequencing data. Previous efforts to measure contamination used loci known to display complete silencing of the maternal allele in the fetal placenta (*i.e., exp_Mat_* = 0), where maternal expression can be assumed to originate exclusively from contaminating cells and *expCont* can be estimated as the difference between *expTotal* and *exp_Pat_* (equation 2). However, these genes represented a small sample of the transcriptome, (N=10 genes) and did not yield consistent estimates of contamination in our analyses, presumably due to variation in the extent of maternal silencing and inconsistent levels of expression across replicates (Finn et al. 2014).

*If exp_Mat_ = 0, then:*

𝑒𝑥𝑝_𝐶𝑜𝑛𝑡_ = 𝑒𝑥𝑝_𝑇𝑜𝑡𝑎𝑙_ – 𝑒𝑥𝑝_𝑃𝑎𝑡_

*Identification of diagnostic loci to measure maternal contamination*

Instead of using maternally silenced genes, we targeted genes with no allelic imbalance in the fetal

placenta, where *exp_Mat_* and *exp_Pat_* can be assumed to be approximately equal, and *exp_Cont_* can be estimated from the deviation from a 1:1 expression ratio of maternal and paternal alleles (equation 3).

*If exp_Mat_ = exp_Pat_, then:*

𝑒𝑥𝑝_𝐶𝑜𝑛𝑡_ = 𝑒𝑥𝑝_𝑇𝑜𝑡𝑎𝑙_ − 2𝑒𝑥𝑝_𝑃𝑎𝑡_

To identify a set of diagnostic genes, we generated a series of predictions on gene expression

patterns consistent with no allelic imbalance in the fetal placenta:

1. *No differences in expression levels in decidua tissue between genetic lines used for a cross.* Differences in gene expression levels between genetic lines could reflect *cis* regulatory variation of parental alleles, which would cause allelic imbalance in embryonic tissues. Thus, genes showing no differential expression in the maternal decidua of both genetic lines used for a cross, were considered less likely to exhibit allelic imbalance in the fetal placenta.
2. *Maternal bias in the junctional zone and no bias in the labyrinth zone.* Our analysis on the transcriptome-wide effects of maternal contamination showed large effects on junctional zone tissue (which is directly juxtaposed to decidual tissue) and no effect on labyrinth zone tissue, therefore, a gene showing maternal bias in the junctional, but not the labyrinth zone, is likely to have no allelic imbalance and show maternal bias solely as a result of contamination.
3. *Allelic expression shifts towards maternal expression in the fetal placenta should be the same in both directions of a reciprocal cross if the decidua to fetal expression ratio conserved between cross directions.* The extent of maternal bias in a gene is proportional to its decidua to fetal expression ratio (D:F). Therefore, we reasoned that genes with no allelic imbalance and with conserved D:F in the fetal placenta in both directions of a reciprocal cross should show consistent levels of maternal bias in both cross directions. We note that this might also be true for imprinted genes showing maternal bias, so we excluded the small subset of known imprinted genes in the mouse fetal placenta from this calculation. We also note that genes with allelic imbalance should not show this pattern given the asymmetry of true allelic proportions between reciprocal crosses (supplementary figure S11).

We identified genes fitting all three predictions in each cross type separately and used them as diagnostic genes to measure maternal contamination in the fetal placenta. To avoid noise incorporated by sampling error on expression and allelic ratios, we filtered our diagnostic gene set excluding genes with low expression levels (TPM < 2) in either the decidua or the fetal placenta and genes with low power for assessing allele specific expression (*i.e.,* less than 2 diagnostic variants between genetic lines).

*Calculating average contamination*

The size of diagnostic gene sets varied by cross type ranging from 169 to 308 with the largest number of diagnostic genes in the cross between the most divergent lines as expected from increased power for allele specific expression. We used these genes to estimate *exp_Cont_* using equation 3 and calculated contamination (*C*) as the average proportion of a gene’s expression level in the decidua (*exp_Dec_)* required to explain maternal contamination in the fetal placenta (equation 4). This value should be consistent across genes and determined by the amount of remnant decidual tissue in a fetal placenta sample.


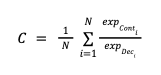


*Accounting for contamination and re-calculating P1 and P2*

We calculated the theoretical value of *expCont* for each gene in the transcriptome as a function of its expression level in the decidua and the estimated average contamination (equation 5). Next, we

accounted for contamination in the transcriptome by subtracting *expCont* from the observed levels of maternal (O.exp_Mat_) and total expression (O.exp_Total_) in the fetal placenta and re-calculated the P1 and P2 values used to estimate parent-of-origin bias score (equations 6 and 7).


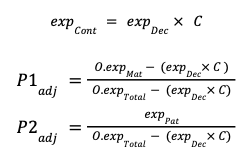


To account for variation in decidual expression levels between genetic lines, estimates of decidual

expression were generated individually for each maternal line. All expression levels were estimated from RNA-seq data and standardized using transcripts per million reads (TPM).

**References**

Finn, Elizabeth H., Cheryl L. Smith, Jesse Rodriguez, Arend Sidow, and Julie C. Baker. 2014.

“Maternal Bias and Escape from X Chromosome Imprinting in the Midgestation Mouse Placenta.”

*Developmental Biology* 390 (1): 80–92. https://doi.org/10.1016/j.ydbio.2014.02.020.

Proudhon, Charlotte, and Déborah Bourc’his. 2010. “Identification and Resolution of Artifacts in the Interpretation of Imprinted Gene Expression.” *Briefings in Functional Genomics* 9 (5–6): 374–84. https://doi.org/10.1093/bfgp/elq020.
